# Crystal structures of four indole derivatives as possible cannabinoid allosteric antagonists

**DOI:** 10.1107/S2056989015008476

**Published:** 2015-05-20

**Authors:** Jamie R. Kerr, Laurent Trembleau, John M. D. Storey, James L. Wardell, William T. A. Harrison

**Affiliations:** a Department of Chemistry, University of Aberdeen, Meston Walk, Aberdeen AB24 3UE, Scotland; b Fundação Oswaldo Cruz, Instituto de Tecnologia em Fármacos-Far Manguinhos, 21041-250 Rio de Janeiro, RJ, Brazil

**Keywords:** crystal structure, indole, cannabin­oid allosteric antagonist, N—H⋯O hydrogen bond

## Abstract

The crystal structures of four indole derivatives with various substituents at the 2-, 3- and 5-positions of the ring system are described, namely, ethyl 3-(5-chloro-2-phenyl-1*H*-indol-3-yl)-3-phenyl­propano­ate, C_25_H_22_ClNO_2_, (I), 2-bromo-3-(2-nitro-1-phenyl­eth­yl)-1*H*-indole, C_16_H_13_BrN_2_O_2_, (II), 5-meth­oxy-3-(2-nitro-1-phenyl­eth­yl)-2-phenyl-1*H*-indole, C_23_H_20_N_2_O_3_, (III), and 5-chloro-3-(2-nitro-1-phenyl­eth­yl)-2-phenyl-1*H*-indole, C_22_H_17_ClN_2_O_2_, (IV). The dominant inter­molecular inter­action in each case is an N—H⋯O hydrogen bond, which generates either chains or inversion dimers. Weak C—H⋯O, C—H⋯π and π–π inter­actions occur in these structures but there is no consistent pattern amongst them. Two of these compounds act as modest enhancers of CB1 cannabanoid signalling and two are inactive.

## Abstract 2

The crystal structures of four indole derivatives with various substituents at the 2-, 3- and 5-positions of the ring system are described. The dominant inter­molecular inter­action in each case is an N—H⋯O hydrogen bond, which generates either chains or inversion dimers. Weak C—H⋯O, C—H⋯π and π–π inter­actions occur in these structures but there is no consistent pattern amongst them. Two of these compounds act as modest enhancers of CB1 cannabanoid signalling and two are inactive.

## Chemical context   

The indole ring system is an important element of many natural and synthetic mol­ecules with important biological activities (Biswal *et al.*, 2012 ▸; Kaushik *et al.*, 2013 ▸; Sharma *et al.*, 2010 ▸). As part of our ongoing studies in this area, a group of indole derivatives with different substituents at the 2, 3 and 5-positions of the ring system were synthesised and tested as possible cannabinoid allosteric antagonists (Kerr, 2013 ▸). These compounds are analogues of 3-(2-nitro-1-phenyl­eth­yl)-2-phenyl-1*H*-indole (known as F087; see scheme), a positive allosteric modulator of CB1 (Adam *et al.*, 2007 ▸).
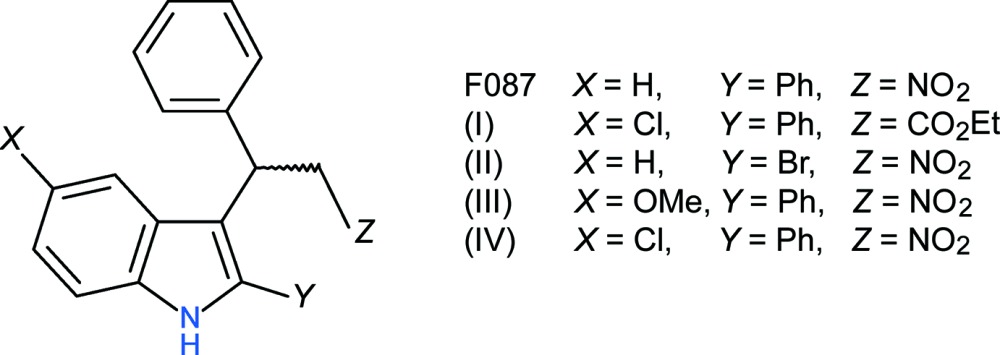


We now report the crystal structures of four of the compounds from that study, *viz*. ethyl 3-(5-chloro-2-phenyl-1*H*-indol-3-yl)-3-phenyl­propano­ate, (I), 2-bromo-3-(2-nitro-1-phenyl­eth­yl)-1*H*-indole, (II), 5-meth­oxy-3-(2-nitro-1-phenyl­eth­yl)-2-phenyl-1*H*-indole, (III), and 5-chloro-3-(2-nitro-1-phenyl­eth­yl)-2-phenyl-1*H*-indole, (IV). Compounds (III) and (IV) were found to act as moderate enhancers of CB1 signalling at 1 µ*M* concentration (Kerr, 2013 ▸) but compounds (I) and (II) were inactive.

## Structural commentary   

Each compound crystallizes in a centrosymmetric space group [*Pbcn* for (I), *P*2_1_/*c* for (II) and *P* 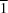 for (III) and (IV)] with one mol­ecule in the asymmetric unit: in each structure, the stereogenic carbon atom (C9) was assigned an arbitrary *R* configuration. All the bond lengths and angles in these compounds lie within their expected ranges and full details are available in the CIF.

**Figure 1:**
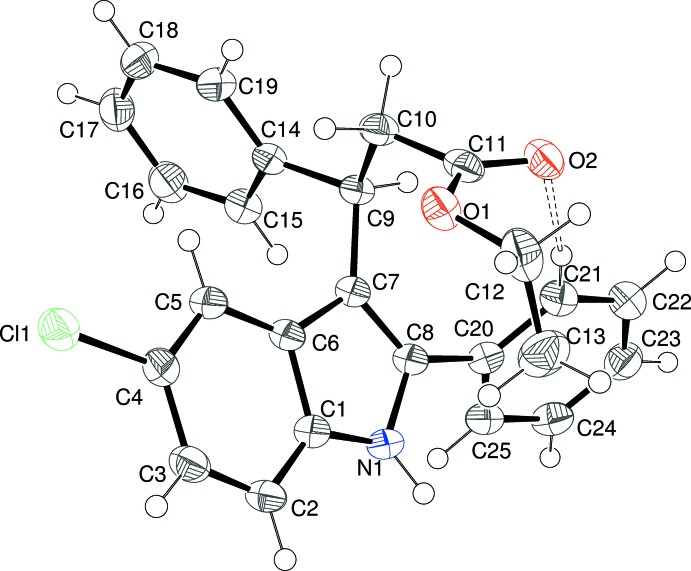
The mol­ecular structure of (I), showing 50% displacement ellipsoids. The double-dashed line indicates a weak C—H⋯O hydrogen bond.

The mol­ecular structure of (I) is illustrated in Fig. 1 ▸. The deviations of atoms Cl1, C9 and C20 from the mean plane (r.m.s. deviation = 0.033 Å) of the indole ring system are 0.0293 (17), −0.156 (2) and −0.008 (2) Å, respectively. The larger deviation for C9 may arise from the steric crowding around it. The dihedral angle between the indole ring system and the C20-phenyl ring is 54.07 (4)° and the C7—C8—C20—C21 torsion angle is 53.7 (3)°. This twisting facilitates the formation of an intra­molecular C—H⋯O inter­action (Table 1 ▸), which generates an *S*(9) ring. Atom H9 is close to eclipsed with C8 (C8—C7—C9—H9 = 2°) and the C14 phenyl ring and the C10-bonded ester groups project to opposite sides of the indole ring, as qu­anti­fied by the C8—C7—C9—C14 and C8—C7—C9—C10 torsion angles of 119.22 (17) and −115.32 (18)°, respectively. Looking down the C9—C7 bond with C8 facing upwards, the C14-phenyl group lies to the left of the indole ring system and the ester group to the right. With respect to the C9—C10 bond, atoms C11 and C14 have an anti disposition [C14—C9—C10—C11 = 175.39 (13)°]. The C11—O1—C12—C13 torsion angle is −81.27 (19)° and the dihedral angle between the indole ring system and the C14 phenyl ring is 86.55 (4)°.

**Figure 2:**
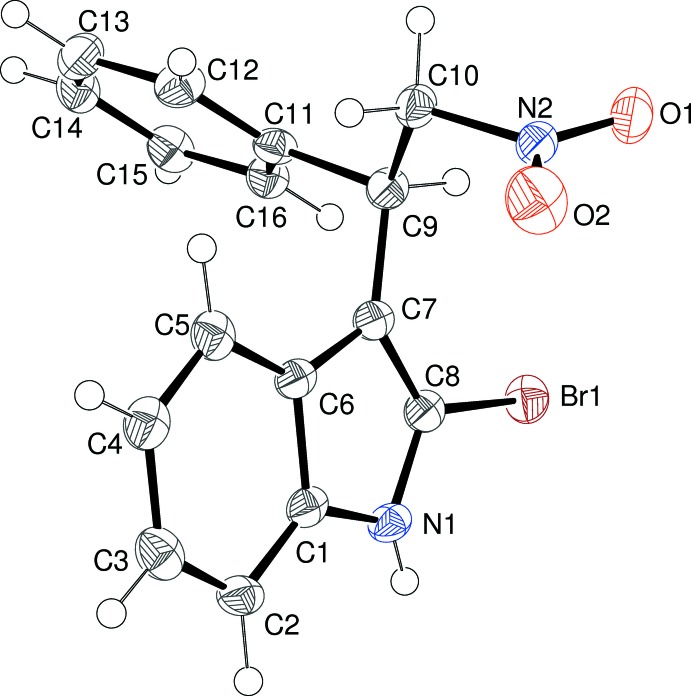
The mol­ecular structure of (II), showing 50% displacement ellipsoids.

The mol­ecular structure of (II) is shown in Fig. 2 ▸. Atoms Br1 and C9 deviate from the mean plane of the indole ring system (r.m.s. deviation = 0.011 Å) by 0.073 (3) and 0.134 (4) Å, respectively. Again, the larger deviation of C9 can be ascribed to steric crowding. The substituents bonded to the 3-position of the ring in (II) are characterized by the C8—C7—C9—H9 torsion angle of −15° and the corresponding C8—C7—C9—C11 and C8—C7—C9—C10 angles of 101.0 (3)° and −134.3 (3)°, respectively. These indicate that the substituents attached to C9 are twisted by about 18° compared to the equivalent groups in (I), although the phenyl ring and nitro group still project in roughly opposite senses with respect to the indole ring. The N2—C10—C9—C11 torsion angle of −174.4 (3)° indicates that the nitro group and phenyl ring lie in an *anti* orientation about the C10—C9 bond. The dihedral angle between the indole ring system and the phenyl ring is 81.69 (7)°.

**Figure 3:**
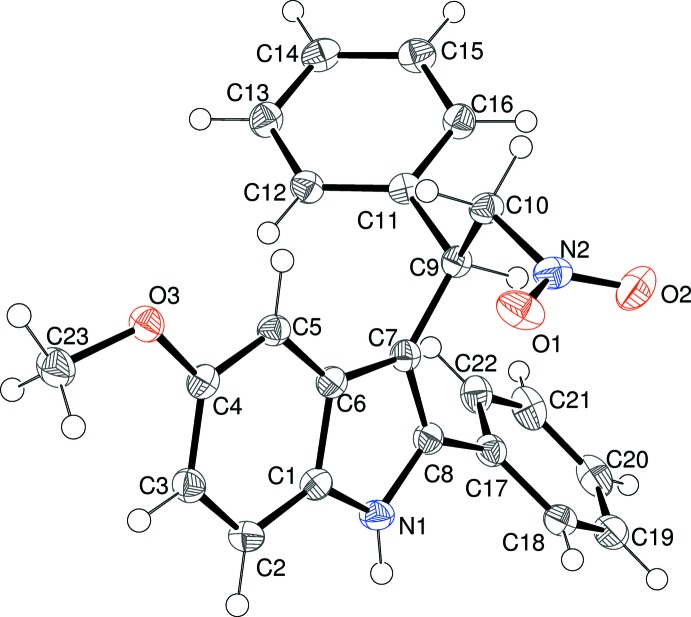
The mol­ecular structure of (III), showing 50% displacement ellipsoids.

Fig. 3 ▸ shows the mol­ecular structure of (III). The r.m.s. deviation for the atoms making up the indole ring system is 0.013Å, and O3, C9 and C17 deviate from the mean plane by 0.0273 (12), −0.1302 (14), and 0.148 (1)Å, respectively. The dihedral angle between the indole ring plane and the C17-ring is 53.76 (3). This is similar to the equivalent value for (I), but the twist is in the opposite sense, as indicated by the C7—C8—C17—C22 torsion angle of −52.40 (15)°: in this case no intra­molecular C—H⋯O bond is present. The dihedral angle between the indole ring and the C11 ring is 67.12 (3)°. The C8—C7—C9—H9, C8—C7—C9—C11 and C8—C7—C9—C10 torsion angles are −17, 102.46 (11) and −133.20 (10)°, respectively, which are almost identical to the corresponding values for (II). These indicate that the C9—H9 bond is twisted away from the indole plane to the same side of the mol­ecule as the nitro group: looking down the C9—C7 bond, C9—H9 is rotated in a clockwise sense with respect to the ring. The disposition of N2 and C11 about the C10—C9 bond is *anti* [torsion angle = −171.63 (8)°]. The methyl C atom of the meth­oxy group deviates from the indole plane by −0.1302 (14) Å, *i.e.* slightly towards the side of the mol­ecule occupied by the C11 phenyl ring.

**Figure 4:**
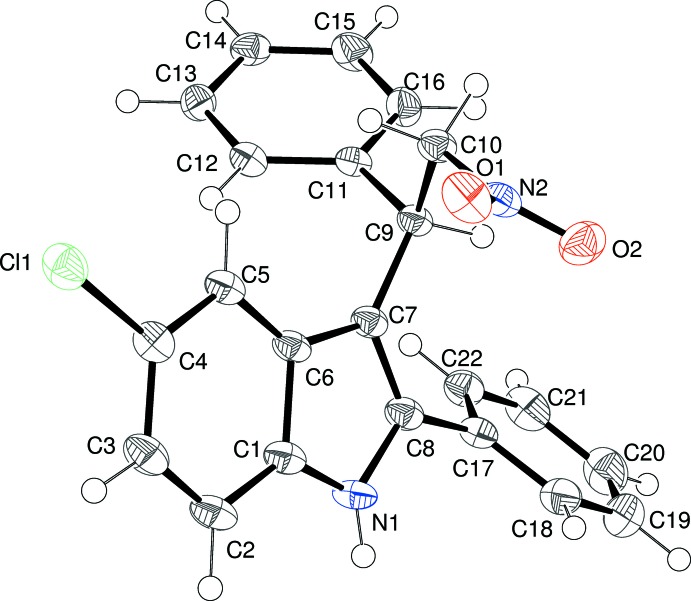
The mol­ecular structure of (IV), showing 50% displacement ellipsoids.

A view of the mol­ecular structure of (IV) can be seen in Fig. 4 ▸. The indole ring system has an r.m.s. deviation of 0.008 Å for its nine non-hydrogen atoms and Cl1, C9 and C17 deviate from the mean plane by 0.009 (1), 0.093 (1) and −0.044 (1)Å. Thus, the displacement of C9 is slightly smaller than in the other three structures presented here. In terms of the orientation of the substituents at the 3-position of the indole ring, the C8—C7—C9—H9, C8—C7—C9—C11 and C8—C7—C9—C10 torsion angles are −17, 102.42 (14) and −133.94 (12)°, respectively, which are very similar to the equivalent data for (II) and (III), again indicating that C9—H9 is twisted towards the nitro group. The N2—C10—C9—C11 torsion angle of 179.61 (9)° shows that the anti orientation of N2 and C11 exactly mirrors that of the equivalent atoms in (II) and (III).

All-in-all, the conformations of (II), (III) and (IV) are very similar, especially in terms of the orientations of the substit­uents attached to C9 with respect to the indole ring. (I) differs slightly in that C9—H9 lies almost in the indole ring plane rather than being twisted away from it, which possibly correlates with the intra­molecular C—H⋯O inter­action noted above. Of course, in every case, crystal symmetry generates an equal number of mol­ecules of the opposite chirality (*i.e.*, *S* configuration of C9), with an anti­clockwise twist of C9—H9 with respect to the indole ring system.

## Supra­molecular features   

As might be expected, the dominant supra­molecular motif in all these compounds involve N—H⋯O hydrogen bonds, although the resulting topologies [chains for (I) and (II) and dimers for (III) and (IV)] are different. Various weak inter­actions also occur, as described below and listed in Tables 1 ▸–4 ▸ ▸ ▸, respectively.

**Figure 5:**
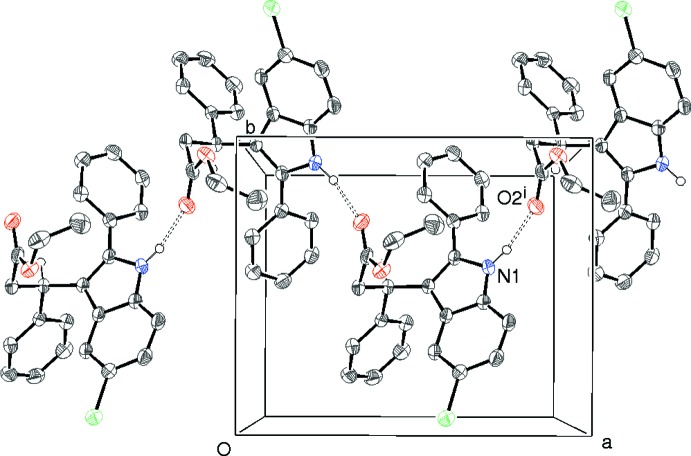
Partial packing diagram for (I), showing the formation of [100] chains linked by N—H⋯O hydrogen bonds (double-dashed lines). Symmetry code as in Table 1 ▸.

In (I), the N1—H1⋯O2^i^ [(i) = 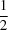 − *x*, *y* − 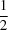, *z*] bond links the mol­ecules into [100] chains with a *C*(8) chain motif (Fig. 5 ▸); adjacent mol­ecules are related by b-glide symmetry. A *PLATON* (Spek, 2009 ▸) analysis of the packing in (I) indicated the presence of no fewer than four C—H⋯π inter­actions, although the C10, C16 and C19 bonds must be very weak based on the long H⋯π separation. Together, these links lead to a three-dimensional network in the crystal. There are no aromatic π–π stacking inter­actions in (I), as the shortest ring centroid–centroid separation is greater than 4.6 Å.

**Figure 6:**
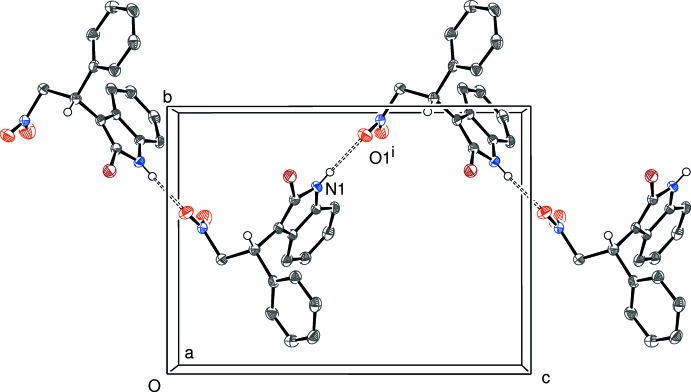
Partial packing diagram for (II), showing the formation of [001] chains linked by N—H⋯O hydrogen bonds (double-dashed lines). Symmetry code as in Table 2 ▸.

The mol­ecules of (II) are linked by N1—H1—O2^i^ [(i) = *x*, 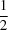 − *y*, *z* − 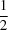] hydrogen bonds into [001] chains (Fig. 6 ▸) characterized by a *C*(8) motif: adjacent mol­ecules are related by *c*-glide symmetry. Just one C—H⋯π inter­action occurs in the crystal of (II) but a π–π stacking inter­action involving inversion-related pairs of C1–C6 benzene rings is also observed: the centroid–centroid separation is 3.7122 (16) Å and the slippage is 1.69 Å. The weak links connect the chains into a three-dimensional network.

**Figure 7:**
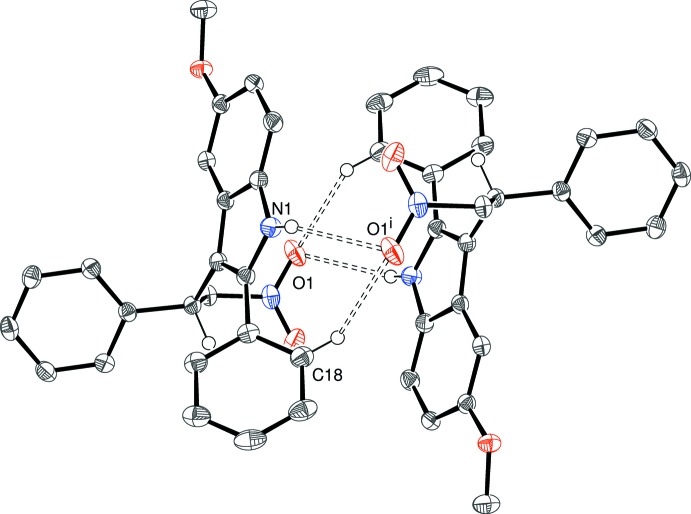
An inversion dimer in the crystal of (III) linked by pairs of N—H⋯O and C—H⋯O hydrogen bonds (double-dashed lines). Symmetry code as in Table 3 ▸.

In (III), inversion dimers linked by N1—H1⋯O1^i^ and N1^i^—H1^i^⋯O1 [(i) = −*x*, 1 − *y*, 1 − *z*] hydrogen bonds occur, which generate 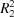
(16) loops. The dimer linkage is reinforced by a pair of C12—H12⋯O1 inter­actions (Fig. 7 ▸). The dimers are linked by several C—H⋯O and C—H⋯π inter­actions, generating a three-dimensional network. The shortest ring centroid–centroid separation is over 4.7 Å.

**Figure 8:**
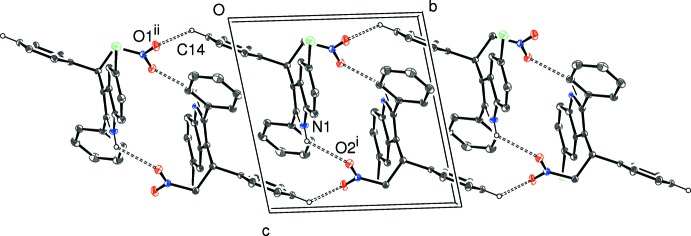
Fragment of an [010] chain in the crystal of (IV) linked by N—H⋯O and C—H⋯O hydrogen bonds (double-dashed lines). Symmetry codes as in Table 4 ▸.

In the crystal of (IV), the mol­ecules associate into inversion dimers linked by N1—H1⋯O2^i^ and N1^i^—H1^i^⋯O2 [(i) = 1 − *x*, 1 − *y*, 1 − *z*] hydrogen bonds (Fig. 8 ▸). Just one weak C—H⋯O hydrogen bond connects the dimers into [010] chains. The shortest ring centroid–centroid separation is over 4.5 Å.

## Database survey   

There are over 4000 indole derivatives with different substituents (including H) at the 2, 3 and 5 positions of the ring system reported in the Cambridge Structural Database (CSD; Groom & Allen, 2014 ▸). Narrowing the survey to indole deriv­atives with a C atom bonded to the 2-position of the ring and an *sp* ^3^-hybridized C atom with two further C atoms and one H atom bonded to it at the 3-position (as per C9 in the present structures) yielded 72 hits. An analysis of the dihedral angle in these structures corresponding to C8—C7—C9—H9 in the present structures showed a wide spread of values with no obvious overall pattern.

## Synthesis and crystallization   

A mixture of sodium chloride (219 mg, 3.75 mmol) and diethyl 2-([5-chloro-2-phenyl-1*H*-indol-3-yl]{phen­yl}meth­yl)malonate (847 mg, 1.78 mmol), [prepared from diethyl benzyl­idene­malonate and 5-chloro-2-phenyl­indole in the presence of Cu(OTf)_2_] in DMSO (10.8 ml) and water (150 ml) was stirred at 443K for 16 h. After cooling to room temperature, water was added until a precipitate formed (25 ml). The mixture was extracted into DCM (3 × 25 ml), washed with saturated NaCl(aq) (15 ml), dried over sodium sulfate, filtered and evaporated to leave a red oil. Flash chromatography (1:1 DCM, hexa­nes) afforded (I) as a colourless solid (638 mg, 89%), m.p. 464K. Colourless blocks were recrystallized from methanol solution at room temperature. IR (Nujol, cm^−1^) 3391, 2911, 1738, 1629, 1581, 1556, 1445, 1399, 1283, 1271, 1215, 1208, 1145, 1113, 1077, 874, 852,761. HRMS (ESI) for C_25_H_23_^35^ClNO_2_ [*M* + H]^+^ calculated 404.1418, found 404.1416.

A mixture of indole (1.069 g, 9.13 mmol), trans-β-nitro­styrene (1.372 g, 9.20 mmol) and sulfamic acid (178 mg, 1.83 mmol) were refluxed in EtOH (45 ml) for 24 h. Removal of the solvent and flash chromatography (1:3 diethyl ether, hexa­nes) afforded 3-(2-nitro-1-phenyl­eth­yl)-1*H*-indole as a colourless solid (2.020 g, 83%). This was refluxed in ClCl_4_ (40 ml) with NBS (1.505 g, 8.46 mmol) for 96 h, filtered and the solvent evaporated under reduced pressure to leave a red oily residue. Flash chromatography of the residue (1:5 EtOAc, hexa­nes) gave (II) as a peach-coloured solid (1.386 g, 53%). Pale-brown plates were recrystallized from methanol solution at room temperature; m.p. 436K; IR (KBr, cm^−1^) 3353, 2987, 2923, 2856, 1548, 1452, 1337, 740 and 701; RMS (ESI) for C_16_H_13_^79^BrN_2_O_2_Na [*M* + Na]^+^ calculated 367.0058, found 367.0049.

A mixture of trans-β-nitro­styrene (167 mg, 1.12 mmol), sulfamic acid (22 mg, 0.22 mmol) and 5-meth­oxy-2-phenyl-1*H*-indole (250 mg, 1.12 mmol), prepared from *p*-meth­oxy­phenyl­hydrazine hydro­chloride, aceto­phenone and PPA in EtOH (5 ml) was stirred at 323K for 40 h. The solvent was removed under reduced pressure and the residue was flash chromatographed (1:5 EtOAc, hexa­nes) to provide (III) as an orange solid (210 mg, 50%): Light-yellow blocks were recrystallized from methanol solution at room temperature; m.p. 434–436K; IR (KBr, cm^−1^) 3407, 1629, 1600, 1581, 1534, 1369, 1200 and 1141; HRMS (ESI) for C_23_H_21_N_2_O_3_ [*M* + H]^+^ calculated 373.1553, found 373.1544.

5-Chloro-2-phenyl-1*H*-indole (1.286 g, 5.65 mmol), trans-β-nitro­styrene (843 mg, 5.65 mmol) and sulfamic acid (110 mg, 1.13 mmol) were stirred in EtOH (80 ml) at reflux for 15 h. The solvent was removed under reduced pressure and the crude product was purified by flash chromatography (1:4 EtOAc, hexa­nes then 1:2 EtOAc, hexa­nes) to give the product as a yellow solid (1.105 g, 52%). R_f_ 0.23 (1:4 EtOAc, hexa­nes); m.p. 457–459K; IR (KBr, cm^−1^) 3396, 3034, 1740, 1598, 1510, 1318, 1055 and 839; HRMS (ESI) for C_22_H_18_N_2_O_2_Cl [*M* + H]^+^ calculated 377.1057, found 377.1054.

## Refinement   

Crystal data, data collection and structure refinement details are summarized in Table 5 ▸. The N-bound H atoms were located in difference maps and their positions freely refined. The C-bound H atoms were geometrically placed (C—H = 0.93–0.98 Å) and refined as riding atoms. The constraint *U*_iso_(H) = 1.2*U*_eq_(carrier) or 1.5*U*_eq_(methyl carrier) was applied in all cases. The methyl H atoms (if any) were allowed to rotate, but not to tip, to best fit the electron density.

## Supplementary Material

Crystal structure: contains datablock(s) I, II, III, IV, global. DOI: 10.1107/S2056989015008476/lh5763sup1.cif

Structure factors: contains datablock(s) I. DOI: 10.1107/S2056989015008476/lh5763Isup2.hkl

Structure factors: contains datablock(s) II. DOI: 10.1107/S2056989015008476/lh5763IIsup3.hkl

Structure factors: contains datablock(s) III. DOI: 10.1107/S2056989015008476/lh5763IIIsup4.hkl

Structure factors: contains datablock(s) IV. DOI: 10.1107/S2056989015008476/lh5763IVsup5.hkl

Supporting information file. DOI: 10.1107/S2056989015008476/lh5763Isup6.cml

Supporting information file. DOI: 10.1107/S2056989015008476/lh5763IIsup7.cml

Supporting information file. DOI: 10.1107/S2056989015008476/lh5763IIIsup8.cml

Supporting information file. DOI: 10.1107/S2056989015008476/lh5763IVsup9.cml

CCDC references: 1062393, 1062392, 1062391, 1062390

Additional supporting information:  crystallographic information; 3D view; checkCIF report

## Figures and Tables

**Table 1:** Hydrogen-bond geometry (Å, °) for (I) *Cg*2 and *Cg*4 are the centroids of the C1–C6 and C20–C25 rings, respectively.

*D*—H⋯*A*	*D*—H	H⋯*A*	*D*⋯*A*	*D*—H⋯*A*
C21—H21⋯O2	0.93	2.34	3.258 (2)	169
N1—H1⋯O2^i^	0.91 (2)	1.95 (2)	2.8310 (18)	163.0 (18)
C10—H10*A*⋯*Cg*4^ii^	0.97	2.93	3.8022 (18)	150
C12—H12*A*⋯*Cg*2^iii^	0.97	2.97	3.702 (2)	133
C16—H16⋯*Cg*4^iv^	0.93	2.78	3.643 (2)	154
C19—H19⋯*Cg*2^i^	0.93	2.96	3.7860 (18)	149

Symmetry codes: (i) 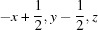; (ii) 
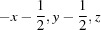
; (iii) 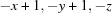; (iv) 
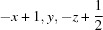
.

**Table 2:** Hydrogen-bond geometry (Å, °) for (II) *Cg*2 and *Cg*4 are the centroids of the C1–C6 ring.

*D*—H⋯*A*	*D*—H	H⋯*A*	*D*⋯*A*	*D*—H⋯*A*
N1—H1⋯O1^i^	0.80 (4)	2.32 (4)	3.087 (3)	161 (4)
C12—H12⋯*Cg*2^ii^	0.95	2.75	3.500 (3)	136

Symmetry codes: (i) 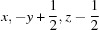; (ii) 
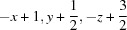
.

**Table 3:** Hydrogen-bond geometry (Å, °) for (III) *Cg*2 and *Cg*4 are the centroids of the C1–C6 and C17–C22 rings, respectively.

*D*—H⋯*A*	*D*—H	H⋯*A*	*D*⋯*A*	*D*—H⋯*A*
N1—H1⋯O1^i^	0.867 (14)	2.470 (14)	3.1872 (13)	140.5 (12)
C10—H10*A*⋯O3^ii^	0.99	2.56	2.9934 (14)	107
C14—H14⋯O3^iii^	0.95	2.51	3.4546 (14)	173
C18—H18⋯O1^i^	0.95	2.59	3.2877 (14)	131
C21—H21⋯*Cg*2^iv^	0.95	2.83	3.5297 (13)	131
C23—H23*C*⋯*Cg*4^v^	0.98	2.76	3.5781 (13)	141

Symmetry codes: (i) 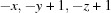; (ii) 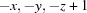; (iii) 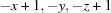; (iv) 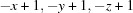; (v) 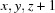.

**Table 4:** Hydrogen-bond geometry (Å, °) for (IV)

*D*—H⋯*A*	*D*—H	H⋯*A*	*D*⋯*A*	*D*—H⋯*A*
N1—H1⋯O2^i^	0.814 (16)	2.517 (16)	3.0806 (15)	127.4 (14)
C14—H14⋯O1^ii^	0.95	2.60	3.1827 (17)	120

Symmetry codes: (i) 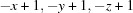; (ii) 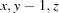.

**Table 5:** Experimental details

	(I)	(II)	(III)	(IV)
Crystal data				
Chemical formula	C_25_H_22_ClNO_2_	C_16_H_13_BrN_2_O_2_	C_23_H_20_N_2_O_3_	C_22_H_17_ClN_2_O_2_
*M*_r_	403.89	345.19	372.41	376.83
Crystal system, space group	Orthorhombic, *P* *b* *c* *n*	Monoclinic, *P*2_1_/*c*	Triclinic, *P* 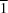	Triclinic, *P* 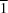
Temperature (K)	100	100	100	100
*a*, *b*, *c* (Å)	10.1558 (7), 12.1446 (9), 33.605 (2)	9.7223 (7), 10.2804 (7), 13.9652 (10)	9.7561 (7), 10.0258 (7), 10.8942 (8)	9.5830 (7), 9.7555 (7), 10.2307 (7)
α, β, γ (°)	90, 90, 90	90, 91.238 (2), 90	116.415 (5), 91.843 (4), 97.963 (4)	79.546 (6), 77.966 (6), 87.455 (7)
*V* (Å^3^)	4144.8 (5)	1395.48 (17)	939.84 (12)	919.87 (11)
*Z*	8	4	2	2
Radiation type	Mo *K*α	Mo *K*α	Mo *K*α	Mo *K*α
μ (mm^−1^)	0.21	2.95	0.09	0.23
Crystal size (mm)	0.22 × 0.19 × 0.07	0.22 × 0.19 × 0.05	0.24 × 0.21 × 0.03	0.48 × 0.36 × 0.16

Data collection				
Diffractometer	Rigaku Mercury CCD	Rigaku Mercury CCD	Rigaku Mercury CCD	Rigaku Mercury CCD
Absorption correction	–	Multi-scan (*SADABS*; Sheldrick, 1996 ▸)	–	Multi-scan (*SADABS*; Sheldrick, 1996 ▸)
*T*_min_, *T*_max_	–	0.563, 0.867	–	0.899, 0.965
No. of measured, independent and observed [*I* > 2σ(*I*)] reflections	27690, 4720, 3714	14919, 3213, 2911	12625, 4305, 3782	13253, 4138, 3363
*R*_int_	0.079	0.042	0.028	0.023
(sin θ/λ)_max_ (Å^−1^)	0.648	0.650	0.650	0.649

Refinement				
*R*[*F* ^2^ > 2σ(*F* ^2^)], *wR*(*F* ^2^), *S*	0.053, 0.153, 1.05	0.040, 0.108, 1.07	0.035, 0.097, 1.06	0.031, 0.085, 1.06
No. of reflections	4720	3213	4305	4138
No. of parameters	266	193	257	247
H-atom treatment	H atoms treated by a mixture of independent and constrained refinement	H atoms treated by a mixture of independent and constrained refinement	H atoms treated by a mixture of independent and constrained refinement	H atoms treated by a mixture of independent and constrained refinement
Δρ_max_, Δρ_min_ (e Å^−3^)	0.54, −0.25	1.26, −0.83	0.30, −0.22	0.27, −0.23

Computer programs: *CrystalClear* (Rigaku, 2012 ▸), *SHELXS97* and *SHELXL97* (Sheldrick, 2008 ▸), *ORTEP-3 for Windows* (Farrugia, 2012 ▸) and *publCIF* (Westrip, 2010 ▸).

## supplementary crystallographic information

### (I) Ethyl 3-(5-chloro-2-phenyl-1H-indol-3-yl)-3-phenylpropanoate Crystal data

**Table:**

C_25_H_22_ClNO_2_	*F*(000) = 1696
*M**_r_* = 403.89	*D*_x_ = 1.294 Mg m^−^^3^
Orthorhombic, *P**b**c**n*	Mo *K*α radiation, λ = 0.71075 Å
Hall symbol: -P 2n 2ab	θ = 2.6–27.5°
*a* = 10.1558 (7) Å	µ = 0.21 mm^−^^1^
*b* = 12.1446 (9) Å	*T* = 100 K
*c* = 33.605 (2) Å	Block, colourless
*V* = 4144.8 (5) Å^3^	0.22 × 0.19 × 0.07 mm
*Z* = 8	

### (I) Ethyl 3-(5-chloro-2-phenyl-1H-indol-3-yl)-3-phenylpropanoate Data collection

**Table:**

Rigaku Mercury CCD diffractometer	3714 reflections with *I* > 2σ(*I*)
Radiation source: fine-focus sealed tube	*R*_int_ = 0.079
Graphite monochromator	θ_max_ = 27.4°, θ_min_ = 2.6°
ω scans	*h* = −10→13
27690 measured reflections	*k* = −15→15
4720 independent reflections	*l* = −27→43

### (I) Ethyl 3-(5-chloro-2-phenyl-1H-indol-3-yl)-3-phenylpropanoate Refinement

**Table:**

Refinement on *F*^2^	Primary atom site location: structure-invariant direct methods
Least-squares matrix: full	Secondary atom site location: difference Fourier map
*R*[*F*^2^ > 2σ(*F*^2^)] = 0.053	Hydrogen site location: inferred from neighbouring sites
*wR*(*F*^2^) = 0.153	H atoms treated by a mixture of independent and constrained refinement
*S* = 1.05	*w* = 1/[σ^2^(*F*_o_^2^) + (0.0961*P*)^2^ + 0.2647*P*] where *P* = (*F*_o_^2^ + 2*F*_c_^2^)/3
4720 reflections	(Δ/σ)_max_ < 0.001
266 parameters	Δρ_max_ = 0.54 e Å^−^^3^
0 restraints	Δρ_min_ = −0.24 e Å^−^^3^

### (I) Ethyl 3-(5-chloro-2-phenyl-1H-indol-3-yl)-3-phenylpropanoate Special details

**Table:**

Geometry. All e.s.d.'s (except the e.s.d. in the dihedral angle between two l.s. planes) are estimated using the full covariance matrix. The cell e.s.d.'s are taken into account individually in the estimation of e.s.d.'s in distances, angles and torsion angles; correlations between e.s.d.'s in cell parameters are only used when they are defined by crystal symmetry. An approximate (isotropic) treatment of cell e.s.d.'s is used for estimating e.s.d.'s involving l.s. planes.
Refinement. Refinement of *F*^2^ against ALL reflections. The weighted *R*-factor *wR* and goodness of fit *S* are based on *F*^2^, conventional *R*-factors *R* are based on *F*, with *F* set to zero for negative *F*^2^. The threshold expression of *F*^2^ > σ(*F*^2^) is used only for calculating *R*-factors(gt) *etc*. and is not relevant to the choice of reflections for refinement. *R*-factors based on *F*^2^ are statistically about twice as large as those based on *F*, and *R*- factors based on ALL data will be even larger.

### (I) Ethyl 3-(5-chloro-2-phenyl-1H-indol-3-yl)-3-phenylpropanoate Fractional atomic coordinates and isotropic or equivalent isotropic displacement parameters (Å^2^)

**Table:**

	*x*	*y*	*z*	*U*_iso_*/*U*_eq_	
C1	0.54948 (15)	0.29890 (13)	0.09779 (5)	0.0311 (4)	
C2	0.61955 (17)	0.22877 (13)	0.07259 (5)	0.0348 (4)	
H2	0.5861	0.1603	0.0654	0.042*	
C3	0.73983 (18)	0.26430 (14)	0.05867 (5)	0.0347 (4)	
H3	0.7883	0.2201	0.0415	0.042*	
C4	0.78952 (16)	0.36789 (14)	0.07051 (5)	0.0326 (4)	
C5	0.72080 (16)	0.43914 (13)	0.09457 (5)	0.0302 (4)	
H5	0.7557	0.5072	0.1016	0.036*	
C6	0.59588 (16)	0.40567 (12)	0.10815 (5)	0.0290 (3)	
C7	0.49436 (15)	0.45698 (12)	0.13159 (5)	0.0281 (3)	
C8	0.39449 (16)	0.38103 (13)	0.13567 (5)	0.0297 (3)	
C9	0.49669 (15)	0.57406 (12)	0.14740 (5)	0.0279 (3)	
H9	0.4144	0.5860	0.1620	0.033*	
C10	0.50018 (16)	0.65784 (13)	0.11262 (5)	0.0313 (4)	
H10A	0.4879	0.7316	0.1231	0.038*	
H10B	0.5857	0.6550	0.0998	0.038*	
C11	0.39491 (17)	0.63402 (13)	0.08240 (5)	0.0318 (4)	
C12	0.3469 (2)	0.55713 (18)	0.01910 (6)	0.0457 (5)	
H12A	0.3883	0.5551	−0.0069	0.055*	
H12B	0.2762	0.6108	0.0182	0.055*	
C13	0.2900 (2)	0.44405 (18)	0.02866 (7)	0.0514 (5)	
H13A	0.2238	0.4257	0.0094	0.077*	
H13B	0.2515	0.4452	0.0547	0.077*	
H13C	0.3590	0.3900	0.0278	0.077*	
C14	0.61049 (16)	0.59166 (12)	0.17675 (5)	0.0293 (4)	
C15	0.62031 (17)	0.52108 (14)	0.20950 (5)	0.0354 (4)	
H15	0.5587	0.4651	0.2126	0.042*	
C16	0.72023 (18)	0.53266 (16)	0.23754 (6)	0.0408 (4)	
H16	0.7253	0.4848	0.2591	0.049*	
C17	0.81262 (18)	0.61658 (16)	0.23304 (6)	0.0415 (4)	
H17	0.8796	0.6250	0.2517	0.050*	
C18	0.80455 (18)	0.68715 (15)	0.20092 (6)	0.0403 (4)	
H18	0.8664	0.7430	0.1980	0.048*	
C19	0.70415 (17)	0.67531 (14)	0.17278 (5)	0.0350 (4)	
H19	0.6996	0.7234	0.1513	0.042*	
C20	0.26765 (16)	0.38379 (13)	0.15729 (5)	0.0312 (4)	
C21	0.17591 (17)	0.46826 (14)	0.15152 (5)	0.0355 (4)	
H21	0.1954	0.5257	0.1342	0.043*	
C22	0.05587 (17)	0.46706 (15)	0.17144 (6)	0.0386 (4)	
H22	−0.0045	0.5234	0.1672	0.046*	
C23	0.02557 (18)	0.38227 (15)	0.19767 (5)	0.0368 (4)	
H23	−0.0548	0.3817	0.2109	0.044*	
C24	0.11659 (17)	0.29816 (14)	0.20392 (5)	0.0363 (4)	
H24	0.0973	0.2416	0.2216	0.044*	
C25	0.23610 (17)	0.29836 (14)	0.18378 (5)	0.0339 (4)	
H25	0.2957	0.2414	0.1879	0.041*	
N1	0.42951 (14)	0.28435 (11)	0.11609 (5)	0.0324 (3)	
H1	0.3687 (19)	0.2319 (17)	0.1108 (6)	0.039*	
O1	0.44406 (12)	0.59120 (10)	0.04889 (4)	0.0357 (3)	
O2	0.27802 (12)	0.65045 (10)	0.08742 (4)	0.0367 (3)	
Cl1	0.94493 (4)	0.40680 (4)	0.052348 (14)	0.04005 (16)	

### (I) Ethyl 3-(5-chloro-2-phenyl-1H-indol-3-yl)-3-phenylpropanoate Atomic displacement parameters (Å^2^)

**Table:**

	*U*^11^	*U*^22^	*U*^33^	*U*^12^	*U*^13^	*U*^23^
C1	0.0296 (8)	0.0234 (7)	0.0403 (9)	0.0003 (6)	−0.0034 (7)	−0.0012 (6)
C2	0.0384 (9)	0.0222 (7)	0.0438 (9)	0.0028 (7)	−0.0050 (7)	−0.0056 (7)
C3	0.0378 (9)	0.0279 (8)	0.0384 (9)	0.0066 (7)	−0.0033 (7)	−0.0055 (7)
C4	0.0291 (8)	0.0288 (8)	0.0400 (9)	0.0027 (7)	−0.0020 (7)	0.0033 (7)
C5	0.0322 (9)	0.0218 (7)	0.0368 (9)	0.0001 (6)	−0.0032 (7)	−0.0004 (6)
C6	0.0305 (8)	0.0203 (7)	0.0362 (9)	0.0024 (6)	−0.0034 (7)	−0.0002 (6)
C7	0.0284 (8)	0.0205 (7)	0.0353 (8)	0.0014 (6)	−0.0024 (6)	0.0001 (6)
C8	0.0305 (8)	0.0214 (7)	0.0371 (8)	0.0015 (6)	−0.0038 (7)	−0.0013 (6)
C9	0.0282 (8)	0.0193 (7)	0.0361 (9)	0.0007 (6)	0.0006 (6)	−0.0003 (6)
C10	0.0333 (9)	0.0211 (7)	0.0393 (9)	0.0001 (6)	0.0021 (7)	0.0021 (6)
C11	0.0368 (9)	0.0208 (7)	0.0378 (9)	0.0050 (7)	0.0013 (7)	0.0033 (6)
C12	0.0416 (10)	0.0577 (12)	0.0379 (10)	0.0153 (9)	−0.0057 (8)	−0.0072 (8)
C13	0.0528 (12)	0.0515 (12)	0.0500 (12)	−0.0075 (10)	−0.0030 (9)	−0.0182 (9)
C14	0.0277 (8)	0.0225 (7)	0.0377 (9)	0.0033 (6)	0.0010 (7)	−0.0047 (6)
C15	0.0336 (9)	0.0286 (8)	0.0439 (10)	0.0017 (7)	−0.0009 (7)	−0.0007 (7)
C16	0.0403 (10)	0.0397 (10)	0.0423 (10)	0.0072 (8)	−0.0026 (8)	−0.0012 (8)
C17	0.0316 (9)	0.0462 (11)	0.0466 (11)	0.0064 (8)	−0.0068 (8)	−0.0120 (8)
C18	0.0313 (9)	0.0345 (9)	0.0551 (11)	−0.0032 (7)	0.0005 (8)	−0.0108 (8)
C19	0.0352 (9)	0.0257 (8)	0.0441 (10)	−0.0017 (7)	0.0003 (7)	−0.0033 (7)
C20	0.0303 (8)	0.0251 (8)	0.0382 (9)	−0.0029 (6)	−0.0030 (7)	−0.0034 (6)
C21	0.0337 (9)	0.0290 (8)	0.0439 (10)	0.0004 (7)	−0.0009 (7)	0.0020 (7)
C22	0.0347 (9)	0.0342 (9)	0.0468 (10)	0.0034 (7)	−0.0016 (7)	−0.0022 (8)
C23	0.0316 (8)	0.0375 (9)	0.0414 (9)	−0.0049 (7)	0.0034 (7)	−0.0071 (7)
C24	0.0414 (10)	0.0283 (8)	0.0392 (9)	−0.0064 (7)	0.0024 (7)	−0.0013 (7)
C25	0.0354 (9)	0.0245 (8)	0.0419 (9)	−0.0013 (7)	0.0002 (7)	−0.0026 (7)
N1	0.0301 (7)	0.0215 (6)	0.0456 (8)	−0.0030 (6)	−0.0016 (6)	−0.0043 (6)
O1	0.0335 (7)	0.0377 (7)	0.0359 (7)	0.0078 (5)	0.0000 (5)	−0.0013 (5)
O2	0.0353 (7)	0.0326 (6)	0.0422 (7)	0.0083 (5)	0.0009 (5)	−0.0009 (5)
Cl1	0.0337 (3)	0.0348 (3)	0.0517 (3)	0.00319 (17)	0.00755 (18)	0.00006 (18)

### (I) Ethyl 3-(5-chloro-2-phenyl-1H-indol-3-yl)-3-phenylpropanoate Geometric parameters (Å, º)

**Table:**

C1—N1	1.376 (2)	C12—H12B	0.9700
C1—C2	1.396 (2)	C13—H13A	0.9600
C1—C6	1.423 (2)	C13—H13B	0.9600
C2—C3	1.377 (3)	C13—H13C	0.9600
C2—H2	0.9300	C14—C19	1.398 (2)
C3—C4	1.413 (2)	C14—C15	1.398 (2)
C3—H3	0.9300	C15—C16	1.392 (3)
C4—C5	1.375 (2)	C15—H15	0.9300
C4—Cl1	1.7569 (18)	C16—C17	1.394 (3)
C5—C6	1.408 (2)	C16—H16	0.9300
C5—H5	0.9300	C17—C18	1.381 (3)
C6—C7	1.439 (2)	C17—H17	0.9300
C7—C8	1.378 (2)	C18—C19	1.398 (3)
C7—C9	1.518 (2)	C18—H18	0.9300
C8—N1	1.392 (2)	C19—H19	0.9300
C8—C20	1.479 (2)	C20—C21	1.399 (2)
C9—C14	1.535 (2)	C20—C25	1.404 (2)
C9—C10	1.550 (2)	C21—C22	1.391 (2)
C9—H9	0.9800	C21—H21	0.9300
C10—C11	1.503 (2)	C22—C23	1.390 (3)
C10—H10A	0.9700	C22—H22	0.9300
C10—H10B	0.9700	C23—C24	1.393 (3)
C11—O2	1.216 (2)	C23—H23	0.9300
C11—O1	1.337 (2)	C24—C25	1.390 (2)
C12—O1	1.465 (2)	C24—H24	0.9300
C12—C13	1.524 (3)	C25—H25	0.9300
C12—H12A	0.9700	N1—H1	0.91 (2)
			
N1—C1—C2	130.09 (15)	C12—C13—H13B	109.5
N1—C1—C6	107.52 (14)	H13A—C13—H13B	109.5
C2—C1—C6	122.38 (16)	C12—C13—H13C	109.5
C3—C2—C1	117.83 (15)	H13A—C13—H13C	109.5
C3—C2—H2	121.1	H13B—C13—H13C	109.5
C1—C2—H2	121.1	C19—C14—C15	118.16 (16)
C2—C3—C4	120.02 (16)	C19—C14—C9	123.52 (15)
C2—C3—H3	120.0	C15—C14—C9	118.31 (14)
C4—C3—H3	120.0	C16—C15—C14	121.52 (17)
C5—C4—C3	123.02 (16)	C16—C15—H15	119.2
C5—C4—Cl1	119.41 (13)	C14—C15—H15	119.2
C3—C4—Cl1	117.56 (13)	C15—C16—C17	119.43 (17)
C4—C5—C6	117.79 (15)	C15—C16—H16	120.3
C4—C5—H5	121.1	C17—C16—H16	120.3
C6—C5—H5	121.1	C18—C17—C16	119.92 (17)
C5—C6—C1	118.83 (15)	C18—C17—H17	120.0
C5—C6—C7	134.25 (14)	C16—C17—H17	120.0
C1—C6—C7	106.92 (14)	C17—C18—C19	120.54 (17)
C8—C7—C6	106.98 (14)	C17—C18—H18	119.7
C8—C7—C9	127.13 (14)	C19—C18—H18	119.7
C6—C7—C9	125.86 (14)	C18—C19—C14	120.43 (17)
C7—C8—N1	109.24 (14)	C18—C19—H19	119.8
C7—C8—C20	132.44 (15)	C14—C19—H19	119.8
N1—C8—C20	118.27 (14)	C21—C20—C25	118.54 (16)
C7—C9—C14	111.54 (12)	C21—C20—C8	121.89 (15)
C7—C9—C10	110.58 (13)	C25—C20—C8	119.56 (15)
C14—C9—C10	112.09 (13)	C22—C21—C20	120.60 (17)
C7—C9—H9	107.5	C22—C21—H21	119.7
C14—C9—H9	107.5	C20—C21—H21	119.7
C10—C9—H9	107.5	C23—C22—C21	120.47 (17)
C11—C10—C9	111.53 (13)	C23—C22—H22	119.8
C11—C10—H10A	109.3	C21—C22—H22	119.8
C9—C10—H10A	109.3	C22—C23—C24	119.46 (17)
C11—C10—H10B	109.3	C22—C23—H23	120.3
C9—C10—H10B	109.3	C24—C23—H23	120.3
H10A—C10—H10B	108.0	C25—C24—C23	120.31 (16)
O2—C11—O1	123.06 (16)	C25—C24—H24	119.8
O2—C11—C10	124.70 (16)	C23—C24—H24	119.8
O1—C11—C10	112.24 (14)	C24—C25—C20	120.62 (16)
O1—C12—C13	111.45 (16)	C24—C25—H25	119.7
O1—C12—H12A	109.3	C20—C25—H25	119.7
C13—C12—H12A	109.3	C1—N1—C8	109.21 (13)
O1—C12—H12B	109.3	C1—N1—H1	127.3 (13)
C13—C12—H12B	109.3	C8—N1—H1	120.8 (13)
H12A—C12—H12B	108.0	C11—O1—C12	115.72 (14)
C12—C13—H13A	109.5		
			
N1—C1—C2—C3	−179.31 (17)	C10—C9—C14—C19	1.3 (2)
C6—C1—C2—C3	2.3 (3)	C7—C9—C14—C15	−54.82 (19)
C1—C2—C3—C4	1.1 (3)	C10—C9—C14—C15	−179.43 (14)
C2—C3—C4—C5	−2.6 (3)	C19—C14—C15—C16	−0.2 (2)
C2—C3—C4—Cl1	178.83 (13)	C9—C14—C15—C16	−179.44 (15)
C3—C4—C5—C6	0.7 (3)	C14—C15—C16—C17	0.2 (3)
Cl1—C4—C5—C6	179.22 (12)	C15—C16—C17—C18	−0.1 (3)
C4—C5—C6—C1	2.6 (2)	C16—C17—C18—C19	0.1 (3)
C4—C5—C6—C7	−176.81 (18)	C17—C18—C19—C14	−0.1 (3)
N1—C1—C6—C5	177.11 (14)	C15—C14—C19—C18	0.1 (2)
C2—C1—C6—C5	−4.2 (2)	C9—C14—C19—C18	179.36 (15)
N1—C1—C6—C7	−3.36 (18)	C7—C8—C20—C21	53.7 (3)
C2—C1—C6—C7	175.36 (16)	N1—C8—C20—C21	−129.19 (18)
C5—C6—C7—C8	−178.78 (18)	C7—C8—C20—C25	−127.7 (2)
C1—C6—C7—C8	1.79 (18)	N1—C8—C20—C25	49.4 (2)
C5—C6—C7—C9	3.1 (3)	C25—C20—C21—C22	−0.4 (3)
C1—C6—C7—C9	−176.37 (15)	C8—C20—C21—C22	178.19 (16)
C6—C7—C8—N1	0.44 (18)	C20—C21—C22—C23	0.5 (3)
C9—C7—C8—N1	178.56 (15)	C21—C22—C23—C24	0.0 (3)
C6—C7—C8—C20	177.73 (17)	C22—C23—C24—C25	−0.7 (3)
C9—C7—C8—C20	−4.1 (3)	C23—C24—C25—C20	0.8 (3)
C8—C7—C9—C14	119.22 (17)	C21—C20—C25—C24	−0.3 (2)
C6—C7—C9—C14	−63.0 (2)	C8—C20—C25—C24	−178.90 (15)
C8—C7—C9—C10	−115.32 (18)	C2—C1—N1—C8	−174.90 (18)
C6—C7—C9—C10	62.5 (2)	C6—C1—N1—C8	3.69 (19)
C7—C9—C10—C11	50.25 (17)	C7—C8—N1—C1	−2.61 (19)
C14—C9—C10—C11	175.39 (13)	C20—C8—N1—C1	179.66 (14)
C9—C10—C11—O2	72.6 (2)	O2—C11—O1—C12	−4.3 (2)
C9—C10—C11—O1	−106.83 (15)	C10—C11—O1—C12	175.18 (14)
C7—C9—C14—C19	125.93 (16)	C13—C12—O1—C11	−81.27 (19)

### (I) Ethyl 3-(5-chloro-2-phenyl-1H-indol-3-yl)-3-phenylpropanoate Hydrogen-bond geometry (Å, º)

Cg2 and Cg4 are the centroids of the C1–C6 and C20–C25 rings, respectively.

**Table:**

*D*—H···*A*	*D*—H	H···*A*	*D*···*A*	*D*—H···*A*
C21—H21···O2	0.93	2.34	3.258 (2)	169
N1—H1···O2^i^	0.91 (2)	1.95 (2)	2.8310 (18)	163.0 (18)
C10—H10*A*···*Cg*4^ii^	0.97	2.93	3.8022 (18)	150
C12—H12*A*···*Cg*2^iii^	0.97	2.97	3.702 (2)	133
C16—H16···*Cg*4^iv^	0.93	2.78	3.643 (2)	154
C19—H19···*Cg*2^i^	0.93	2.96	3.7860 (18)	149

Symmetry codes: (i) −*x*+1/2, *y*−1/2, *z*; (ii) −*x*−1/2, *y*−1/2, *z*; (iii) −*x*+1, −*y*+1, −*z*; (iv) −*x*+1, *y*, −*z*+1/2.

### (II) 2-Bromo-3-(2-nitro-1-phenylethyl)-1H-indole Crystal data

**Table:**

C_16_H_13_BrN_2_O_2_	*F*(000) = 696
*M**_r_* = 345.19	*D*_x_ = 1.643 Mg m^−^^3^
Monoclinic, *P*2_1_/*c*	Mo *K*α radiation, λ = 0.71075 Å
Hall symbol: -P 2ybc	Cell parameters from 14875 reflections
*a* = 9.7223 (7) Å	θ = 2.9–27.5°
*b* = 10.2804 (7) Å	µ = 2.95 mm^−^^1^
*c* = 13.9652 (10) Å	*T* = 100 K
β = 91.238 (2)°	Slab, pale brown
*V* = 1395.48 (17) Å^3^	0.22 × 0.19 × 0.05 mm
*Z* = 4	

### (II) 2-Bromo-3-(2-nitro-1-phenylethyl)-1H-indole Data collection

**Table:**

Rigaku Mercury CCD diffractometer	3213 independent reflections
Radiation source: fine-focus sealed tube	2911 reflections with *I* > 2σ(*I*)
Graphite monochromator	*R*_int_ = 0.042
ω scans	θ_max_ = 27.5°, θ_min_ = 2.9°
Absorption correction: multi-scan (*SADABS*; Sheldrick, 1996)	*h* = −12→12
*T*_min_ = 0.563, *T*_max_ = 0.867	*k* = −13→13
14919 measured reflections	*l* = −18→17

### (II) 2-Bromo-3-(2-nitro-1-phenylethyl)-1H-indole Refinement

**Table:**

Refinement on *F*^2^	Primary atom site location: structure-invariant direct methods
Least-squares matrix: full	Secondary atom site location: difference Fourier map
*R*[*F*^2^ > 2σ(*F*^2^)] = 0.040	Hydrogen site location: inferred from neighbouring sites
*wR*(*F*^2^) = 0.108	H atoms treated by a mixture of independent and constrained refinement
*S* = 1.07	*w* = 1/[σ^2^(*F*_o_^2^) + (0.0534*P*)^2^ + 2.3689*P*] where *P* = (*F*_o_^2^ + 2*F*_c_^2^)/3
3213 reflections	(Δ/σ)_max_ = 0.001
193 parameters	Δρ_max_ = 1.26 e Å^−^^3^
0 restraints	Δρ_min_ = −0.82 e Å^−^^3^

### (II) 2-Bromo-3-(2-nitro-1-phenylethyl)-1H-indole Special details

**Table:**

Geometry. All e.s.d.'s (except the e.s.d. in the dihedral angle between two l.s. planes) are estimated using the full covariance matrix. The cell e.s.d.'s are taken into account individually in the estimation of e.s.d.'s in distances, angles and torsion angles; correlations between e.s.d.'s in cell parameters are only used when they are defined by crystal symmetry. An approximate (isotropic) treatment of cell e.s.d.'s is used for estimating e.s.d.'s involving l.s. planes.
Refinement. Refinement of *F*^2^ against ALL reflections. The weighted *R*-factor *wR* and goodness of fit *S* are based on *F*^2^, conventional *R*-factors *R* are based on *F*, with *F* set to zero for negative *F*^2^. The threshold expression of *F*^2^ > σ(*F*^2^) is used only for calculating *R*-factors(gt) *etc*. and is not relevant to the choice of reflections for refinement. *R*-factors based on *F*^2^ are statistically about twice as large as those based on *F*, and *R*- factors based on ALL data will be even larger.

### (II) 2-Bromo-3-(2-nitro-1-phenylethyl)-1H-indole Fractional atomic coordinates and isotropic or equivalent isotropic displacement parameters (Å^2^)

**Table:**

	*x*	*y*	*z*	*U*_iso_*/*U*_eq_	
C1	0.4952 (3)	0.3822 (3)	0.59411 (18)	0.0218 (5)	
C2	0.3700 (3)	0.3784 (3)	0.54275 (19)	0.0261 (6)	
H2	0.3510	0.3120	0.4970	0.031*	
C3	0.2753 (3)	0.4750 (3)	0.5611 (2)	0.0297 (6)	
H3	0.1888	0.4745	0.5281	0.036*	
C4	0.3051 (3)	0.5740 (3)	0.6280 (2)	0.0281 (6)	
H4	0.2376	0.6386	0.6395	0.034*	
C5	0.4299 (3)	0.5801 (3)	0.6776 (2)	0.0257 (6)	
H5	0.4492	0.6489	0.7213	0.031*	
C6	0.5274 (3)	0.4817 (2)	0.66142 (18)	0.0210 (5)	
C7	0.6650 (3)	0.4559 (3)	0.69787 (18)	0.0215 (5)	
C8	0.7066 (3)	0.3462 (3)	0.65211 (19)	0.0223 (5)	
C9	0.7553 (3)	0.5367 (3)	0.76399 (19)	0.0229 (5)	
H9	0.8379	0.4833	0.7817	0.027*	
C10	0.6852 (3)	0.5750 (3)	0.85699 (19)	0.0248 (6)	
H10A	0.7475	0.6310	0.8960	0.030*	
H10B	0.6003	0.6249	0.8420	0.030*	
C11	0.8057 (3)	0.6574 (3)	0.70989 (18)	0.0219 (5)	
C12	0.7453 (3)	0.7795 (3)	0.7152 (2)	0.0284 (6)	
H12	0.6706	0.7933	0.7568	0.034*	
C13	0.7938 (3)	0.8824 (3)	0.6596 (2)	0.0313 (6)	
H13	0.7520	0.9657	0.6639	0.038*	
C14	0.9025 (3)	0.8638 (3)	0.5982 (2)	0.0294 (6)	
H14	0.9354	0.9338	0.5607	0.035*	
C15	0.9624 (3)	0.7418 (3)	0.5922 (2)	0.0281 (6)	
H15	1.0363	0.7278	0.5499	0.034*	
C16	0.9147 (3)	0.6396 (3)	0.6479 (2)	0.0247 (5)	
H16	0.9570	0.5566	0.6436	0.030*	
N1	0.6058 (3)	0.2984 (2)	0.59133 (16)	0.0230 (5)	
H1	0.623 (4)	0.242 (3)	0.554 (3)	0.028*	
N2	0.6503 (3)	0.4554 (2)	0.91218 (16)	0.0271 (5)	
O1	0.7431 (2)	0.3967 (2)	0.95513 (16)	0.0339 (5)	
O2	0.5300 (3)	0.4220 (3)	0.91282 (19)	0.0464 (6)	
Br1	0.87595 (3)	0.26053 (3)	0.66349 (2)	0.02856 (12)	

### (II) 2-Bromo-3-(2-nitro-1-phenylethyl)-1H-indole Atomic displacement parameters (Å^2^)

**Table:**

	*U*^11^	*U*^22^	*U*^33^	*U*^12^	*U*^13^	*U*^23^
C1	0.0294 (13)	0.0189 (12)	0.0174 (11)	−0.0045 (10)	0.0064 (10)	0.0008 (9)
C2	0.0357 (15)	0.0257 (13)	0.0169 (12)	−0.0090 (11)	0.0020 (11)	−0.0003 (10)
C3	0.0280 (14)	0.0373 (16)	0.0237 (14)	−0.0035 (12)	0.0021 (11)	0.0056 (12)
C4	0.0321 (15)	0.0271 (14)	0.0255 (14)	0.0026 (11)	0.0075 (12)	0.0026 (11)
C5	0.0289 (14)	0.0279 (14)	0.0205 (13)	−0.0011 (11)	0.0059 (11)	0.0042 (10)
C6	0.0279 (13)	0.0190 (12)	0.0164 (11)	−0.0039 (10)	0.0062 (10)	0.0002 (9)
C7	0.0286 (13)	0.0206 (12)	0.0155 (11)	−0.0043 (10)	0.0058 (10)	0.0007 (9)
C8	0.0266 (13)	0.0222 (12)	0.0184 (12)	−0.0009 (10)	0.0058 (10)	0.0005 (10)
C9	0.0272 (13)	0.0219 (12)	0.0198 (12)	−0.0006 (10)	0.0053 (10)	−0.0005 (10)
C10	0.0336 (15)	0.0199 (12)	0.0210 (13)	0.0006 (11)	0.0041 (11)	0.0007 (10)
C11	0.0238 (12)	0.0246 (13)	0.0172 (12)	−0.0053 (10)	0.0016 (10)	−0.0021 (10)
C12	0.0313 (15)	0.0280 (14)	0.0263 (14)	−0.0014 (12)	0.0078 (11)	−0.0037 (12)
C13	0.0379 (16)	0.0232 (14)	0.0329 (15)	0.0001 (12)	0.0020 (13)	−0.0002 (12)
C14	0.0319 (15)	0.0314 (15)	0.0250 (14)	−0.0079 (12)	0.0010 (11)	0.0074 (11)
C15	0.0242 (13)	0.0368 (16)	0.0235 (14)	−0.0051 (11)	0.0044 (11)	0.0029 (11)
C16	0.0249 (13)	0.0267 (13)	0.0226 (13)	−0.0024 (11)	0.0024 (10)	0.0012 (11)
N1	0.0310 (12)	0.0192 (11)	0.0191 (11)	−0.0020 (9)	0.0053 (9)	−0.0030 (8)
N2	0.0453 (15)	0.0204 (11)	0.0158 (10)	−0.0012 (10)	0.0063 (10)	0.0003 (9)
O1	0.0419 (12)	0.0265 (10)	0.0335 (11)	0.0044 (9)	0.0015 (9)	0.0070 (9)
O2	0.0381 (13)	0.0549 (16)	0.0463 (15)	−0.0109 (12)	0.0043 (11)	0.0110 (12)
Br1	0.03170 (18)	0.02778 (17)	0.02638 (18)	0.00571 (11)	0.00434 (12)	0.00019 (10)

### (II) 2-Bromo-3-(2-nitro-1-phenylethyl)-1H-indole Geometric parameters (Å, º)

**Table:**

C1—N1	1.379 (4)	C9—H9	1.0000
C1—C2	1.399 (4)	C10—N2	1.495 (3)
C1—C6	1.419 (4)	C10—H10A	0.9900
C2—C3	1.382 (4)	C10—H10B	0.9900
C2—H2	0.9500	C11—C12	1.388 (4)
C3—C4	1.408 (4)	C11—C16	1.395 (4)
C3—H3	0.9500	C12—C13	1.400 (4)
C4—C5	1.385 (4)	C12—H12	0.9500
C4—H4	0.9500	C13—C14	1.388 (4)
C5—C6	1.408 (4)	C13—H13	0.9500
C5—H5	0.9500	C14—C15	1.387 (4)
C6—C7	1.445 (4)	C14—H14	0.9500
C7—C8	1.363 (4)	C15—C16	1.392 (4)
C7—C9	1.510 (4)	C15—H15	0.9500
C8—N1	1.373 (4)	C16—H16	0.9500
C8—Br1	1.871 (3)	N1—H1	0.80 (4)
C9—C10	1.531 (4)	N2—O2	1.219 (4)
C9—C11	1.538 (4)	N2—O1	1.231 (3)
			
N1—C1—C2	129.7 (2)	N2—C10—C9	109.6 (2)
N1—C1—C6	107.9 (2)	N2—C10—H10A	109.7
C2—C1—C6	122.5 (3)	C9—C10—H10A	109.7
C3—C2—C1	117.4 (3)	N2—C10—H10B	109.7
C3—C2—H2	121.3	C9—C10—H10B	109.7
C1—C2—H2	121.3	H10A—C10—H10B	108.2
C2—C3—C4	121.0 (3)	C12—C11—C16	118.7 (3)
C2—C3—H3	119.5	C12—C11—C9	124.3 (2)
C4—C3—H3	119.5	C16—C11—C9	117.0 (2)
C5—C4—C3	122.0 (3)	C11—C12—C13	120.4 (3)
C5—C4—H4	119.0	C11—C12—H12	119.8
C3—C4—H4	119.0	C13—C12—H12	119.8
C4—C5—C6	118.2 (3)	C14—C13—C12	120.5 (3)
C4—C5—H5	120.9	C14—C13—H13	119.7
C6—C5—H5	120.9	C12—C13—H13	119.7
C5—C6—C1	118.9 (3)	C15—C14—C13	119.3 (3)
C5—C6—C7	134.1 (3)	C15—C14—H14	120.4
C1—C6—C7	106.9 (2)	C13—C14—H14	120.4
C8—C7—C6	105.6 (2)	C14—C15—C16	120.2 (3)
C8—C7—C9	124.6 (3)	C14—C15—H15	119.9
C6—C7—C9	129.5 (2)	C16—C15—H15	119.9
C7—C8—N1	111.7 (2)	C15—C16—C11	121.0 (3)
C7—C8—Br1	128.3 (2)	C15—C16—H16	119.5
N1—C8—Br1	120.0 (2)	C11—C16—H16	119.5
C7—C9—C10	113.4 (2)	C8—N1—C1	107.8 (2)
C7—C9—C11	109.3 (2)	C8—N1—H1	121 (3)
C10—C9—C11	111.2 (2)	C1—N1—H1	130 (3)
C7—C9—H9	107.6	O2—N2—O1	123.5 (3)
C10—C9—H9	107.6	O2—N2—C10	117.6 (3)
C11—C9—H9	107.6	O1—N2—C10	118.8 (3)
			
N1—C1—C2—C3	178.9 (3)	C6—C7—C9—C11	−71.6 (3)
C6—C1—C2—C3	−1.3 (4)	C7—C9—C10—N2	62.0 (3)
C1—C2—C3—C4	0.9 (4)	C11—C9—C10—N2	−174.4 (2)
C2—C3—C4—C5	0.6 (4)	C7—C9—C11—C12	97.6 (3)
C3—C4—C5—C6	−1.6 (4)	C10—C9—C11—C12	−28.4 (4)
C4—C5—C6—C1	1.2 (4)	C7—C9—C11—C16	−78.9 (3)
C4—C5—C6—C7	179.5 (3)	C10—C9—C11—C16	155.2 (2)
N1—C1—C6—C5	−179.9 (2)	C16—C11—C12—C13	−0.3 (4)
C2—C1—C6—C5	0.2 (4)	C9—C11—C12—C13	−176.7 (3)
N1—C1—C6—C7	1.3 (3)	C11—C12—C13—C14	0.2 (5)
C2—C1—C6—C7	−178.5 (2)	C12—C13—C14—C15	0.2 (5)
C5—C6—C7—C8	−178.4 (3)	C13—C14—C15—C16	−0.6 (4)
C1—C6—C7—C8	0.0 (3)	C14—C15—C16—C11	0.5 (4)
C5—C6—C7—C9	−4.7 (5)	C12—C11—C16—C15	0.0 (4)
C1—C6—C7—C9	173.8 (2)	C9—C11—C16—C15	176.6 (3)
C6—C7—C8—N1	−1.4 (3)	C7—C8—N1—C1	2.3 (3)
C9—C7—C8—N1	−175.6 (2)	Br1—C8—N1—C1	−177.72 (18)
C6—C7—C8—Br1	178.61 (19)	C2—C1—N1—C8	177.6 (3)
C9—C7—C8—Br1	4.5 (4)	C6—C1—N1—C8	−2.2 (3)
C8—C7—C9—C10	−134.3 (3)	C9—C10—N2—O2	−105.5 (3)
C6—C7—C9—C10	53.0 (4)	C9—C10—N2—O1	75.3 (3)
C8—C7—C9—C11	101.0 (3)		

### (II) 2-Bromo-3-(2-nitro-1-phenylethyl)-1H-indole Hydrogen-bond geometry (Å, º)

Cg2 and Cg4 are the centroids of the C1–C6 ring.

**Table:**

*D*—H···*A*	*D*—H	H···*A*	*D*···*A*	*D*—H···*A*
N1—H1···O1^i^	0.80 (4)	2.32 (4)	3.087 (3)	161 (4)
C12—H12···*Cg*2^ii^	0.95	2.75	3.500 (3)	136

Symmetry codes: (i) *x*, −*y*+1/2, *z*−1/2; (ii) −*x*+1, *y*+1/2, −*z*+3/2.

### (III) 5-Methoxy-3-(2-nitro-1-phenylethyl)-2-phenyl-1H-indole Crystal data

**Table:**

C_23_H_20_N_2_O_3_	*Z* = 2
*M**_r_* = 372.41	*F*(000) = 392
Triclinic, *P*1	*D*_x_ = 1.316 Mg m^−^^3^
Hall symbol: -P 1	Mo *K*α radiation, λ = 0.71075 Å
*a* = 9.7561 (7) Å	Cell parameters from 12105 reflections
*b* = 10.0258 (7) Å	θ = 2.9–27.5°
*c* = 10.8942 (8) Å	µ = 0.09 mm^−^^1^
α = 116.415 (5)°	*T* = 100 K
β = 91.843 (4)°	Slab, light yellow
γ = 97.963 (4)°	0.24 × 0.21 × 0.03 mm
*V* = 939.84 (12) Å^3^	

### (III) 5-Methoxy-3-(2-nitro-1-phenylethyl)-2-phenyl-1H-indole Data collection

**Table:**

Rigaku Mercury CCD diffractometer	3782 reflections with *I* > 2σ(*I*)
Radiation source: fine-focus sealed tube	*R*_int_ = 0.028
Graphite monochromator	θ_max_ = 27.5°, θ_min_ = 2.9°
ω scans	*h* = −12→12
12625 measured reflections	*k* = −13→13
4305 independent reflections	*l* = −14→14

### (III) 5-Methoxy-3-(2-nitro-1-phenylethyl)-2-phenyl-1H-indole Refinement

**Table:**

Refinement on *F*^2^	Primary atom site location: structure-invariant direct methods
Least-squares matrix: full	Secondary atom site location: difference Fourier map
*R*[*F*^2^ > 2σ(*F*^2^)] = 0.035	Hydrogen site location: inferred from neighbouring sites
*wR*(*F*^2^) = 0.097	H atoms treated by a mixture of independent and constrained refinement
*S* = 1.06	*w* = 1/[σ^2^(*F*_o_^2^) + (0.0492*P*)^2^ + 0.1954*P*] where *P* = (*F*_o_^2^ + 2*F*_c_^2^)/3
4305 reflections	(Δ/σ)_max_ = 0.001
257 parameters	Δρ_max_ = 0.30 e Å^−^^3^
0 restraints	Δρ_min_ = −0.22 e Å^−^^3^

### (III) 5-Methoxy-3-(2-nitro-1-phenylethyl)-2-phenyl-1H-indole Special details

**Table:**

Geometry. All e.s.d.'s (except the e.s.d. in the dihedral angle between two l.s. planes) are estimated using the full covariance matrix. The cell e.s.d.'s are taken into account individually in the estimation of e.s.d.'s in distances, angles and torsion angles; correlations between e.s.d.'s in cell parameters are only used when they are defined by crystal symmetry. An approximate (isotropic) treatment of cell e.s.d.'s is used for estimating e.s.d.'s involving l.s. planes.
Refinement. Refinement of *F*^2^ against ALL reflections. The weighted *R*-factor *wR* and goodness of fit *S* are based on *F*^2^, conventional *R*-factors *R* are based on *F*, with *F* set to zero for negative *F*^2^. The threshold expression of *F*^2^ > σ(*F*^2^) is used only for calculating *R*-factors(gt) *etc*. and is not relevant to the choice of reflections for refinement. *R*-factors based on *F*^2^ are statistically about twice as large as those based on *F*, and *R*- factors based on ALL data will be even larger.

### (III) 5-Methoxy-3-(2-nitro-1-phenylethyl)-2-phenyl-1H-indole Fractional atomic coordinates and isotropic or equivalent isotropic displacement parameters (Å^2^)

**Table:**

	*x*	*y*	*z*	*U*_iso_*/*U*_eq_	
C1	0.19835 (10)	0.50476 (11)	0.67418 (11)	0.0204 (2)	
C2	0.17538 (11)	0.56338 (12)	0.81213 (11)	0.0230 (2)	
H2	0.1769	0.6686	0.8661	0.028*	
C3	0.15006 (11)	0.46425 (12)	0.86931 (11)	0.0218 (2)	
H3	0.1334	0.5015	0.9633	0.026*	
C4	0.14906 (10)	0.30892 (11)	0.78826 (11)	0.0199 (2)	
C5	0.17195 (10)	0.25007 (11)	0.65095 (10)	0.0196 (2)	
H5	0.1709	0.1448	0.5979	0.024*	
C6	0.19683 (10)	0.34839 (11)	0.59073 (10)	0.0191 (2)	
C7	0.22732 (10)	0.33046 (11)	0.45606 (10)	0.0191 (2)	
C8	0.24758 (10)	0.47341 (11)	0.46426 (10)	0.0203 (2)	
C9	0.22632 (10)	0.18956 (11)	0.32343 (10)	0.0191 (2)	
H9	0.2138	0.2182	0.2473	0.023*	
C10	0.10313 (10)	0.06333 (11)	0.29937 (11)	0.0209 (2)	
H10A	0.1067	−0.0259	0.2104	0.025*	
H10B	0.1097	0.0324	0.3736	0.025*	
C11	0.35712 (10)	0.11774 (11)	0.30199 (11)	0.0197 (2)	
C12	0.45009 (11)	0.13848 (12)	0.41090 (11)	0.0222 (2)	
H12	0.4337	0.2004	0.5031	0.027*	
C13	0.56717 (11)	0.06865 (12)	0.38520 (12)	0.0246 (2)	
H13	0.6302	0.0831	0.4601	0.030*	
C14	0.59239 (11)	−0.02169 (12)	0.25117 (12)	0.0258 (2)	
H14	0.6726	−0.0687	0.2342	0.031*	
C15	0.49987 (12)	−0.04312 (12)	0.14192 (12)	0.0263 (2)	
H15	0.5166	−0.1049	0.0498	0.032*	
C16	0.38279 (11)	0.02591 (12)	0.16739 (11)	0.0238 (2)	
H16	0.3195	0.0104	0.0923	0.029*	
C17	0.29178 (11)	0.52239 (11)	0.36018 (11)	0.0216 (2)	
C18	0.21895 (12)	0.61533 (12)	0.32766 (11)	0.0263 (2)	
H18	0.1360	0.6427	0.3682	0.032*	
C19	0.26838 (14)	0.66735 (13)	0.23588 (12)	0.0324 (3)	
H19	0.2195	0.7313	0.2146	0.039*	
C20	0.38861 (14)	0.62652 (13)	0.17520 (12)	0.0333 (3)	
H20	0.4218	0.6627	0.1126	0.040*	
C21	0.46049 (12)	0.53318 (14)	0.20555 (12)	0.0309 (3)	
H21	0.5424	0.5046	0.1632	0.037*	
C22	0.41268 (11)	0.48128 (13)	0.29814 (11)	0.0255 (2)	
H22	0.4623	0.4177	0.3193	0.031*	
C23	0.13008 (14)	0.25934 (13)	0.98450 (11)	0.0301 (3)	
H23A	0.1179	0.1737	1.0062	0.045*	
H23B	0.0556	0.3185	1.0194	0.045*	
H23C	0.2204	0.3235	1.0280	0.045*	
N1	0.22919 (10)	0.57768 (10)	0.59438 (9)	0.02257 (19)	
H1	0.2353 (14)	0.6738 (16)	0.6209 (14)	0.027*	
N2	−0.03129 (9)	0.11860 (10)	0.29782 (10)	0.0245 (2)	
O1	−0.09938 (8)	0.14343 (9)	0.39586 (9)	0.0325 (2)	
O2	−0.06497 (9)	0.13940 (10)	0.19906 (10)	0.0348 (2)	
O3	0.12460 (8)	0.20475 (8)	0.83947 (7)	0.02322 (17)	

### (III) 5-Methoxy-3-(2-nitro-1-phenylethyl)-2-phenyl-1H-indole Atomic displacement parameters (Å^2^)

**Table:**

	*U*^11^	*U*^22^	*U*^33^	*U*^12^	*U*^13^	*U*^23^
C1	0.0195 (5)	0.0209 (5)	0.0220 (5)	0.0045 (4)	0.0026 (4)	0.0104 (4)
C2	0.0250 (5)	0.0197 (5)	0.0228 (5)	0.0063 (4)	0.0036 (4)	0.0075 (4)
C3	0.0213 (5)	0.0246 (5)	0.0191 (5)	0.0062 (4)	0.0039 (4)	0.0088 (4)
C4	0.0161 (4)	0.0225 (5)	0.0229 (5)	0.0030 (4)	0.0022 (4)	0.0120 (4)
C5	0.0178 (5)	0.0188 (4)	0.0216 (5)	0.0037 (3)	0.0025 (4)	0.0085 (4)
C6	0.0156 (4)	0.0207 (5)	0.0204 (5)	0.0038 (3)	0.0020 (4)	0.0087 (4)
C7	0.0165 (4)	0.0208 (5)	0.0201 (5)	0.0031 (3)	0.0021 (4)	0.0094 (4)
C8	0.0186 (5)	0.0212 (5)	0.0205 (5)	0.0037 (4)	0.0012 (4)	0.0088 (4)
C9	0.0172 (4)	0.0207 (5)	0.0200 (5)	0.0036 (4)	0.0026 (4)	0.0098 (4)
C10	0.0173 (5)	0.0199 (5)	0.0251 (5)	0.0045 (4)	0.0029 (4)	0.0093 (4)
C11	0.0180 (4)	0.0196 (4)	0.0227 (5)	0.0030 (3)	0.0046 (4)	0.0104 (4)
C12	0.0199 (5)	0.0217 (5)	0.0229 (5)	0.0022 (4)	0.0022 (4)	0.0086 (4)
C13	0.0184 (5)	0.0256 (5)	0.0292 (6)	0.0018 (4)	−0.0007 (4)	0.0126 (5)
C14	0.0191 (5)	0.0258 (5)	0.0353 (6)	0.0062 (4)	0.0074 (4)	0.0153 (5)
C15	0.0271 (5)	0.0277 (5)	0.0256 (5)	0.0085 (4)	0.0100 (4)	0.0120 (5)
C16	0.0235 (5)	0.0275 (5)	0.0221 (5)	0.0061 (4)	0.0044 (4)	0.0122 (4)
C17	0.0233 (5)	0.0191 (5)	0.0204 (5)	−0.0008 (4)	−0.0005 (4)	0.0086 (4)
C18	0.0343 (6)	0.0205 (5)	0.0218 (5)	0.0052 (4)	0.0003 (4)	0.0074 (4)
C19	0.0514 (7)	0.0217 (5)	0.0236 (6)	0.0034 (5)	−0.0024 (5)	0.0111 (4)
C20	0.0446 (7)	0.0286 (6)	0.0234 (6)	−0.0094 (5)	−0.0017 (5)	0.0136 (5)
C21	0.0266 (5)	0.0369 (6)	0.0249 (6)	−0.0061 (5)	0.0013 (4)	0.0135 (5)
C22	0.0220 (5)	0.0283 (5)	0.0253 (5)	−0.0006 (4)	−0.0006 (4)	0.0129 (5)
C23	0.0429 (7)	0.0261 (5)	0.0213 (5)	0.0033 (5)	0.0039 (5)	0.0117 (5)
N1	0.0283 (5)	0.0177 (4)	0.0223 (4)	0.0047 (3)	0.0042 (4)	0.0093 (4)
N2	0.0186 (4)	0.0191 (4)	0.0321 (5)	0.0024 (3)	0.0020 (4)	0.0087 (4)
O1	0.0209 (4)	0.0300 (4)	0.0343 (5)	0.0038 (3)	0.0078 (3)	0.0036 (4)
O2	0.0278 (4)	0.0361 (5)	0.0484 (5)	0.0061 (3)	−0.0020 (4)	0.0265 (4)
O3	0.0286 (4)	0.0217 (4)	0.0199 (4)	0.0019 (3)	0.0031 (3)	0.0106 (3)

### (III) 5-Methoxy-3-(2-nitro-1-phenylethyl)-2-phenyl-1H-indole Geometric parameters (Å, º)

**Table:**

C1—N1	1.3791 (13)	C13—C14	1.3860 (16)
C1—C2	1.3880 (14)	C13—H13	0.9500
C1—C6	1.4174 (14)	C14—C15	1.3883 (16)
C2—C3	1.3891 (14)	C14—H14	0.9500
C2—H2	0.9500	C15—C16	1.3889 (15)
C3—C4	1.4060 (14)	C15—H15	0.9500
C3—H3	0.9500	C16—H16	0.9500
C4—C5	1.3814 (14)	C17—C22	1.3986 (15)
C4—O3	1.3846 (12)	C17—C18	1.3999 (15)
C5—C6	1.4072 (14)	C18—C19	1.3891 (16)
C5—H5	0.9500	C18—H18	0.9500
C6—C7	1.4428 (13)	C19—C20	1.3865 (19)
C7—C8	1.3811 (14)	C19—H19	0.9500
C7—C9	1.5042 (14)	C20—C21	1.3853 (18)
C8—N1	1.3728 (14)	C20—H20	0.9500
C8—C17	1.4768 (14)	C21—C22	1.3914 (15)
C9—C11	1.5250 (14)	C21—H21	0.9500
C9—C10	1.5421 (13)	C22—H22	0.9500
C9—H9	1.0000	C23—O3	1.4198 (13)
C10—N2	1.4951 (13)	C23—H23A	0.9800
C10—H10A	0.9900	C23—H23B	0.9800
C10—H10B	0.9900	C23—H23C	0.9800
C11—C12	1.3901 (15)	N1—H1	0.867 (14)
C11—C16	1.3954 (15)	N2—O1	1.2243 (12)
C12—C13	1.3933 (15)	N2—O2	1.2267 (13)
C12—H12	0.9500		
			
N1—C1—C2	129.92 (9)	C14—C13—H13	119.8
N1—C1—C6	107.75 (9)	C12—C13—H13	119.8
C2—C1—C6	122.32 (9)	C13—C14—C15	119.66 (10)
C1—C2—C3	118.32 (9)	C13—C14—H14	120.2
C1—C2—H2	120.8	C15—C14—H14	120.2
C3—C2—H2	120.8	C14—C15—C16	119.93 (10)
C2—C3—C4	120.11 (9)	C14—C15—H15	120.0
C2—C3—H3	119.9	C16—C15—H15	120.0
C4—C3—H3	119.9	C15—C16—C11	120.75 (10)
C5—C4—O3	115.49 (9)	C15—C16—H16	119.6
C5—C4—C3	121.77 (9)	C11—C16—H16	119.6
O3—C4—C3	122.74 (9)	C22—C17—C18	119.44 (10)
C4—C5—C6	119.03 (9)	C22—C17—C8	119.24 (9)
C4—C5—H5	120.5	C18—C17—C8	121.24 (10)
C6—C5—H5	120.5	C19—C18—C17	119.78 (11)
C5—C6—C1	118.44 (9)	C19—C18—H18	120.1
C5—C6—C7	134.84 (9)	C17—C18—H18	120.1
C1—C6—C7	106.69 (9)	C20—C19—C18	120.42 (11)
C8—C7—C6	106.53 (9)	C20—C19—H19	119.8
C8—C7—C9	122.90 (9)	C18—C19—H19	119.8
C6—C7—C9	130.34 (9)	C21—C20—C19	120.20 (10)
N1—C8—C7	109.81 (9)	C21—C20—H20	119.9
N1—C8—C17	120.54 (9)	C19—C20—H20	119.9
C7—C8—C17	129.53 (9)	C20—C21—C22	119.95 (11)
C7—C9—C11	116.98 (8)	C20—C21—H21	120.0
C7—C9—C10	113.02 (8)	C22—C21—H21	120.0
C11—C9—C10	106.58 (8)	C21—C22—C17	120.20 (11)
C7—C9—H9	106.5	C21—C22—H22	119.9
C11—C9—H9	106.5	C17—C22—H22	119.9
C10—C9—H9	106.5	O3—C23—H23A	109.5
N2—C10—C9	109.91 (8)	O3—C23—H23B	109.5
N2—C10—H10A	109.7	H23A—C23—H23B	109.5
C9—C10—H10A	109.7	O3—C23—H23C	109.5
N2—C10—H10B	109.7	H23A—C23—H23C	109.5
C9—C10—H10B	109.7	H23B—C23—H23C	109.5
H10A—C10—H10B	108.2	C8—N1—C1	109.21 (9)
C12—C11—C16	119.01 (9)	C8—N1—H1	124.8 (9)
C12—C11—C9	122.66 (9)	C1—N1—H1	126.0 (9)
C16—C11—C9	118.32 (9)	O1—N2—O2	124.00 (10)
C11—C12—C13	120.17 (10)	O1—N2—C10	118.40 (9)
C11—C12—H12	119.9	O2—N2—C10	117.58 (9)
C13—C12—H12	119.9	C4—O3—C23	118.32 (8)
C14—C13—C12	120.48 (10)		
			
N1—C1—C2—C3	−178.02 (10)	C10—C9—C11—C16	75.88 (11)
C6—C1—C2—C3	−0.03 (16)	C16—C11—C12—C13	0.21 (15)
C1—C2—C3—C4	0.44 (15)	C9—C11—C12—C13	179.28 (9)
C2—C3—C4—C5	−0.41 (16)	C11—C12—C13—C14	0.17 (15)
C2—C3—C4—O3	179.94 (9)	C12—C13—C14—C15	−0.27 (15)
O3—C4—C5—C6	179.63 (8)	C13—C14—C15—C16	0.00 (16)
C3—C4—C5—C6	−0.04 (15)	C14—C15—C16—C11	0.38 (16)
C4—C5—C6—C1	0.44 (14)	C12—C11—C16—C15	−0.48 (15)
C4—C5—C6—C7	178.27 (10)	C9—C11—C16—C15	−179.60 (9)
N1—C1—C6—C5	177.97 (9)	N1—C8—C17—C22	123.13 (11)
C2—C1—C6—C5	−0.42 (15)	C7—C8—C17—C22	−52.40 (15)
N1—C1—C6—C7	−0.42 (11)	N1—C8—C17—C18	−53.62 (14)
C2—C1—C6—C7	−178.81 (9)	C7—C8—C17—C18	130.85 (12)
C5—C6—C7—C8	−177.12 (11)	C22—C17—C18—C19	−0.89 (16)
C1—C6—C7—C8	0.88 (11)	C8—C17—C18—C19	175.85 (10)
C5—C6—C7—C9	8.44 (19)	C17—C18—C19—C20	0.67 (17)
C1—C6—C7—C9	−173.56 (10)	C18—C19—C20—C21	0.07 (17)
C6—C7—C8—N1	−1.04 (11)	C19—C20—C21—C22	−0.58 (17)
C9—C7—C8—N1	173.92 (9)	C20—C21—C22—C17	0.34 (17)
C6—C7—C8—C17	174.87 (10)	C18—C17—C22—C21	0.39 (16)
C9—C7—C8—C17	−10.17 (17)	C8—C17—C22—C21	−176.42 (10)
C8—C7—C9—C11	102.46 (11)	C7—C8—N1—C1	0.80 (12)
C6—C7—C9—C11	−83.89 (13)	C17—C8—N1—C1	−175.54 (9)
C8—C7—C9—C10	−133.20 (10)	C2—C1—N1—C8	178.01 (10)
C6—C7—C9—C10	40.46 (14)	C6—C1—N1—C8	−0.21 (11)
C7—C9—C10—N2	58.52 (11)	C9—C10—N2—O1	−108.24 (10)
C11—C9—C10—N2	−171.63 (8)	C9—C10—N2—O2	70.21 (11)
C7—C9—C11—C12	24.35 (13)	C5—C4—O3—C23	166.65 (9)
C10—C9—C11—C12	−103.20 (10)	C3—C4—O3—C23	−13.68 (14)
C7—C9—C11—C16	−156.57 (9)		

### (III) 5-Methoxy-3-(2-nitro-1-phenylethyl)-2-phenyl-1H-indole Hydrogen-bond geometry (Å, º)

Cg2 and Cg4 are the centroids of the C1–C6 and C17–C22 rings, respectively.

**Table:**

*D*—H···*A*	*D*—H	H···*A*	*D*···*A*	*D*—H···*A*
N1—H1···O1^i^	0.867 (14)	2.470 (14)	3.1872 (13)	140.5 (12)
C10—H10*A*···O3^ii^	0.99	2.56	2.9934 (14)	107
C14—H14···O3^iii^	0.95	2.51	3.4546 (14)	173
C18—H18···O1^i^	0.95	2.59	3.2877 (14)	131
C21—H21···*Cg*2^iv^	0.95	2.83	3.5297 (13)	131
C23—H23*C*···*Cg*4^v^	0.98	2.76	3.5781 (13)	141

Symmetry codes: (i) −*x*, −*y*+1, −*z*+1; (ii) −*x*, −*y*, −*z*+1; (iii) −*x*+1, −*y*, −*z*+1; (iv) −*x*+1, −*y*+1, −*z*+1; (v) *x*, *y*, *z*+1.

### (IV) 5-Chloro-3-(2-nitro-1-phenylethyl)-2-phenyl-1H-indole Crystal data

**Table:**

C_22_H_17_ClN_2_O_2_	*V* = 919.87 (11) Å^3^
*M**_r_* = 376.83	*Z* = 2
Triclinic, *P*1	*F*(000) = 392
Hall symbol: -P 1	*D*_x_ = 1.360 Mg m^−^^3^
*a* = 9.5830 (7) Å	Mo *K*α radiation, λ = 0.71073 Å
*b* = 9.7555 (7) Å	µ = 0.23 mm^−^^1^
*c* = 10.2307 (7) Å	*T* = 100 K
α = 79.546 (6)°	Block, colourless
β = 77.966 (6)°	0.48 × 0.36 × 0.16 mm
γ = 87.455 (7)°	

### (IV) 5-Chloro-3-(2-nitro-1-phenylethyl)-2-phenyl-1H-indole Data collection

**Table:**

Rigaku Mercury CCD diffractometer	4138 independent reflections
Radiation source: fine-focus sealed tube	3363 reflections with *I* > 2σ(*I*)
Graphite monochromator	*R*_int_ = 0.023
ω scans	θ_max_ = 27.5°, θ_min_ = 2.1°
Absorption correction: multi-scan (*SADABS*; Sheldrick, 1996)	*h* = −12→12
*T*_min_ = 0.899, *T*_max_ = 0.965	*k* = −11→12
13253 measured reflections	*l* = −13→13

### (IV) 5-Chloro-3-(2-nitro-1-phenylethyl)-2-phenyl-1H-indole Refinement

**Table:**

Refinement on *F*^2^	Primary atom site location: structure-invariant direct methods
Least-squares matrix: full	Secondary atom site location: difference Fourier map
*R*[*F*^2^ > 2σ(*F*^2^)] = 0.031	Hydrogen site location: inferred from neighbouring sites
*wR*(*F*^2^) = 0.085	H atoms treated by a mixture of independent and constrained refinement
*S* = 1.06	*w* = 1/[σ^2^(*F*_o_^2^) + (0.044*P*)^2^ + 0.1384*P*] where *P* = (*F*_o_^2^ + 2*F*_c_^2^)/3
4138 reflections	(Δ/σ)_max_ = 0.002
247 parameters	Δρ_max_ = 0.27 e Å^−^^3^
0 restraints	Δρ_min_ = −0.23 e Å^−^^3^

### (IV) 5-Chloro-3-(2-nitro-1-phenylethyl)-2-phenyl-1H-indole Special details

**Table:**

Geometry. All e.s.d.'s (except the e.s.d. in the dihedral angle between two l.s. planes) are estimated using the full covariance matrix. The cell e.s.d.'s are taken into account individually in the estimation of e.s.d.'s in distances, angles and torsion angles; correlations between e.s.d.'s in cell parameters are only used when they are defined by crystal symmetry. An approximate (isotropic) treatment of cell e.s.d.'s is used for estimating e.s.d.'s involving l.s. planes.
Refinement. Refinement of *F*^2^ against ALL reflections. The weighted *R*-factor *wR* and goodness of fit *S* are based on *F*^2^, conventional *R*-factors *R* are based on *F*, with *F* set to zero for negative *F*^2^. The threshold expression of *F*^2^ > σ(*F*^2^) is used only for calculating *R*-factors(gt) *etc*. and is not relevant to the choice of reflections for refinement. *R*-factors based on *F*^2^ are statistically about twice as large as those based on *F*, and *R*- factors based on ALL data will be even larger.

### (IV) 5-Chloro-3-(2-nitro-1-phenylethyl)-2-phenyl-1H-indole Fractional atomic coordinates and isotropic or equivalent isotropic displacement parameters (Å^2^)

**Table:**

	*x*	*y*	*z*	*U*_iso_*/*U*_eq_	
C1	0.30134 (13)	0.30487 (13)	0.47178 (13)	0.0244 (3)	
C2	0.15631 (13)	0.33679 (14)	0.49919 (14)	0.0293 (3)	
H2	0.1067	0.3398	0.5893	0.035*	
C3	0.08662 (13)	0.36395 (14)	0.39233 (14)	0.0293 (3)	
H3	−0.0124	0.3858	0.4077	0.035*	
C4	0.16322 (13)	0.35909 (14)	0.26078 (13)	0.0259 (3)	
C5	0.30654 (12)	0.32723 (13)	0.23105 (13)	0.0232 (3)	
H5	0.3547	0.3250	0.1404	0.028*	
C6	0.37928 (12)	0.29823 (12)	0.33886 (12)	0.0213 (2)	
C7	0.52344 (12)	0.26002 (12)	0.35141 (12)	0.0208 (2)	
C8	0.52687 (13)	0.24560 (13)	0.48676 (13)	0.0234 (3)	
C9	0.65601 (12)	0.24850 (13)	0.24431 (12)	0.0206 (2)	
H9	0.7395	0.2586	0.2863	0.025*	
C10	0.66676 (13)	0.36530 (13)	0.12008 (12)	0.0230 (3)	
H10A	0.7613	0.3610	0.0593	0.028*	
H10B	0.5927	0.3523	0.0689	0.028*	
C11	0.67609 (12)	0.11141 (13)	0.19138 (12)	0.0205 (2)	
C12	0.56223 (13)	0.03868 (13)	0.17345 (12)	0.0232 (3)	
H12	0.4676	0.0721	0.1988	0.028*	
C13	0.58560 (13)	−0.08258 (13)	0.11871 (13)	0.0253 (3)	
H13	0.5070	−0.1314	0.1065	0.030*	
C14	0.72269 (14)	−0.13258 (13)	0.08191 (13)	0.0260 (3)	
H14	0.7385	−0.2150	0.0435	0.031*	
C15	0.83681 (14)	−0.06224 (14)	0.10125 (15)	0.0300 (3)	
H15	0.9311	−0.0971	0.0774	0.036*	
C16	0.81368 (13)	0.05913 (14)	0.15540 (14)	0.0275 (3)	
H16	0.8925	0.1072	0.1681	0.033*	
C17	0.64585 (13)	0.20526 (14)	0.55687 (12)	0.0243 (3)	
C18	0.68391 (14)	0.28733 (15)	0.64173 (14)	0.0308 (3)	
H18	0.6360	0.3733	0.6516	0.037*	
C19	0.79159 (16)	0.24370 (18)	0.71180 (16)	0.0402 (4)	
H19	0.8166	0.2994	0.7704	0.048*	
C20	0.86283 (16)	0.11925 (18)	0.69676 (16)	0.0414 (4)	
H20	0.9364	0.0898	0.7453	0.050*	
C21	0.82741 (14)	0.03761 (16)	0.61152 (15)	0.0355 (3)	
H21	0.8768	−0.0476	0.6011	0.043*	
C22	0.71956 (13)	0.08033 (14)	0.54118 (13)	0.0278 (3)	
H22	0.6956	0.0245	0.4821	0.033*	
N1	0.39365 (11)	0.27288 (12)	0.55865 (11)	0.0263 (2)	
H1	0.3714 (16)	0.2700 (17)	0.6404 (17)	0.032*	
N2	0.64715 (11)	0.50447 (11)	0.16313 (11)	0.0258 (2)	
O1	0.55567 (10)	0.58312 (10)	0.12206 (11)	0.0354 (2)	
O2	0.72277 (11)	0.53298 (10)	0.23676 (10)	0.0357 (2)	
Cl1	0.07128 (3)	0.39630 (4)	0.12679 (3)	0.03265 (10)	

### (IV) 5-Chloro-3-(2-nitro-1-phenylethyl)-2-phenyl-1H-indole Atomic displacement parameters (Å^2^)

**Table:**

	*U*^11^	*U*^22^	*U*^33^	*U*^12^	*U*^13^	*U*^23^
C1	0.0251 (6)	0.0227 (7)	0.0221 (6)	0.0022 (5)	0.0018 (5)	−0.0036 (5)
C2	0.0257 (6)	0.0306 (7)	0.0269 (7)	0.0039 (5)	0.0054 (5)	−0.0059 (6)
C3	0.0212 (6)	0.0281 (7)	0.0343 (7)	0.0043 (5)	0.0023 (5)	−0.0051 (6)
C4	0.0243 (6)	0.0230 (7)	0.0285 (6)	0.0019 (5)	−0.0038 (5)	−0.0020 (5)
C5	0.0224 (6)	0.0215 (6)	0.0224 (6)	0.0005 (5)	0.0016 (5)	−0.0029 (5)
C6	0.0214 (5)	0.0182 (6)	0.0216 (6)	0.0012 (4)	0.0025 (4)	−0.0043 (5)
C7	0.0209 (5)	0.0189 (6)	0.0210 (6)	0.0012 (4)	0.0004 (4)	−0.0046 (5)
C8	0.0239 (6)	0.0205 (6)	0.0233 (6)	0.0012 (4)	0.0012 (5)	−0.0046 (5)
C9	0.0195 (5)	0.0210 (6)	0.0199 (6)	0.0006 (4)	−0.0001 (4)	−0.0044 (5)
C10	0.0242 (6)	0.0207 (6)	0.0222 (6)	0.0007 (5)	0.0007 (5)	−0.0055 (5)
C11	0.0223 (5)	0.0198 (6)	0.0170 (5)	0.0007 (4)	0.0002 (4)	−0.0023 (5)
C12	0.0212 (5)	0.0223 (6)	0.0235 (6)	0.0013 (4)	−0.0010 (4)	−0.0017 (5)
C13	0.0277 (6)	0.0227 (7)	0.0248 (6)	−0.0037 (5)	−0.0048 (5)	−0.0028 (5)
C14	0.0337 (7)	0.0197 (6)	0.0229 (6)	0.0004 (5)	−0.0003 (5)	−0.0056 (5)
C15	0.0243 (6)	0.0281 (7)	0.0357 (7)	0.0049 (5)	0.0016 (5)	−0.0106 (6)
C16	0.0211 (6)	0.0267 (7)	0.0354 (7)	0.0000 (5)	−0.0015 (5)	−0.0120 (6)
C17	0.0235 (6)	0.0269 (7)	0.0190 (6)	−0.0017 (5)	0.0011 (4)	−0.0010 (5)
C18	0.0306 (7)	0.0329 (8)	0.0275 (7)	−0.0015 (5)	−0.0004 (5)	−0.0075 (6)
C19	0.0373 (8)	0.0517 (10)	0.0345 (8)	−0.0071 (7)	−0.0100 (6)	−0.0105 (7)
C20	0.0312 (7)	0.0536 (10)	0.0386 (8)	−0.0008 (7)	−0.0132 (6)	0.0009 (7)
C21	0.0293 (7)	0.0344 (8)	0.0379 (8)	0.0037 (6)	−0.0047 (6)	0.0026 (6)
C22	0.0286 (6)	0.0260 (7)	0.0263 (6)	0.0001 (5)	−0.0023 (5)	−0.0021 (5)
N1	0.0257 (5)	0.0324 (6)	0.0179 (5)	0.0043 (4)	0.0025 (4)	−0.0055 (5)
N2	0.0279 (5)	0.0211 (6)	0.0242 (5)	−0.0030 (4)	0.0049 (4)	−0.0039 (4)
O1	0.0314 (5)	0.0234 (5)	0.0466 (6)	0.0044 (4)	−0.0006 (4)	−0.0034 (4)
O2	0.0465 (6)	0.0291 (6)	0.0326 (5)	−0.0025 (4)	−0.0066 (4)	−0.0100 (4)
Cl1	0.02364 (15)	0.0386 (2)	0.03372 (18)	0.00338 (12)	−0.00654 (12)	−0.00124 (14)

### (IV) 5-Chloro-3-(2-nitro-1-phenylethyl)-2-phenyl-1H-indole Geometric parameters (Å, º)

**Table:**

C1—N1	1.3683 (17)	C12—C13	1.3885 (18)
C1—C2	1.3921 (17)	C12—H12	0.9500
C1—C6	1.4197 (16)	C13—C14	1.3817 (18)
C2—C3	1.3766 (19)	C13—H13	0.9500
C2—H2	0.9500	C14—C15	1.3828 (19)
C3—C4	1.4002 (18)	C14—H14	0.9500
C3—H3	0.9500	C15—C16	1.3862 (19)
C4—C5	1.3775 (16)	C15—H15	0.9500
C4—Cl1	1.7556 (13)	C16—H16	0.9500
C5—C6	1.4044 (17)	C17—C18	1.3922 (19)
C5—H5	0.9500	C17—C22	1.3991 (19)
C6—C7	1.4404 (16)	C18—C19	1.385 (2)
C7—C8	1.3734 (17)	C18—H18	0.9500
C7—C9	1.5096 (15)	C19—C20	1.384 (2)
C8—N1	1.3751 (15)	C19—H19	0.9500
C8—C17	1.4724 (17)	C20—C21	1.382 (2)
C9—C11	1.5216 (17)	C20—H20	0.9500
C9—C10	1.5344 (17)	C21—C22	1.3866 (19)
C9—H9	1.0000	C21—H21	0.9500
C10—N2	1.4941 (16)	C22—H22	0.9500
C10—H10A	0.9900	N1—H1	0.814 (16)
C10—H10B	0.9900	N2—O2	1.2213 (14)
C11—C12	1.3881 (17)	N2—O1	1.2291 (14)
C11—C16	1.3929 (16)		
			
N1—C1—C2	129.73 (12)	C11—C12—C13	120.50 (11)
N1—C1—C6	107.57 (10)	C11—C12—H12	119.8
C2—C1—C6	122.70 (12)	C13—C12—H12	119.8
C3—C2—C1	118.29 (12)	C14—C13—C12	120.29 (11)
C3—C2—H2	120.9	C14—C13—H13	119.9
C1—C2—H2	120.9	C12—C13—H13	119.9
C2—C3—C4	119.30 (11)	C13—C14—C15	119.74 (12)
C2—C3—H3	120.4	C13—C14—H14	120.1
C4—C3—H3	120.4	C15—C14—H14	120.1
C5—C4—C3	123.55 (12)	C14—C15—C16	120.03 (12)
C5—C4—Cl1	118.42 (10)	C14—C15—H15	120.0
C3—C4—Cl1	118.03 (10)	C16—C15—H15	120.0
C4—C5—C6	117.99 (11)	C15—C16—C11	120.73 (12)
C4—C5—H5	121.0	C15—C16—H16	119.6
C6—C5—H5	121.0	C11—C16—H16	119.6
C5—C6—C1	118.18 (10)	C18—C17—C22	119.25 (12)
C5—C6—C7	135.24 (11)	C18—C17—C8	121.17 (12)
C1—C6—C7	106.59 (11)	C22—C17—C8	119.56 (12)
C8—C7—C6	106.72 (10)	C19—C18—C17	120.07 (14)
C8—C7—C9	122.33 (11)	C19—C18—H18	120.0
C6—C7—C9	130.71 (11)	C17—C18—H18	120.0
C7—C8—N1	109.55 (11)	C20—C19—C18	120.24 (14)
C7—C8—C17	129.83 (11)	C20—C19—H19	119.9
N1—C8—C17	120.61 (11)	C18—C19—H19	119.9
C7—C9—C11	115.84 (10)	C21—C20—C19	120.30 (14)
C7—C9—C10	112.82 (10)	C21—C20—H20	119.8
C11—C9—C10	106.89 (9)	C19—C20—H20	119.8
C7—C9—H9	106.9	C20—C21—C22	119.85 (14)
C11—C9—H9	106.9	C20—C21—H21	120.1
C10—C9—H9	106.9	C22—C21—H21	120.1
N2—C10—C9	110.47 (10)	C21—C22—C17	120.27 (13)
N2—C10—H10A	109.6	C21—C22—H22	119.9
C9—C10—H10A	109.6	C17—C22—H22	119.9
N2—C10—H10B	109.6	C1—N1—C8	109.57 (11)
C9—C10—H10B	109.6	C1—N1—H1	124.3 (11)
H10A—C10—H10B	108.1	C8—N1—H1	126.1 (11)
C12—C11—C16	118.70 (11)	O2—N2—O1	124.12 (11)
C12—C11—C9	122.24 (10)	O2—N2—C10	118.09 (11)
C16—C11—C9	119.02 (11)	O1—N2—C10	117.78 (11)
			
N1—C1—C2—C3	−179.28 (14)	C7—C9—C11—C16	−145.63 (12)
C6—C1—C2—C3	−0.5 (2)	C10—C9—C11—C16	87.68 (13)
C1—C2—C3—C4	−0.2 (2)	C16—C11—C12—C13	−1.00 (18)
C2—C3—C4—C5	0.5 (2)	C9—C11—C12—C13	176.86 (11)
C2—C3—C4—Cl1	−179.27 (11)	C11—C12—C13—C14	0.26 (18)
C3—C4—C5—C6	0.0 (2)	C12—C13—C14—C15	0.75 (19)
Cl1—C4—C5—C6	179.72 (10)	C13—C14—C15—C16	−1.0 (2)
C4—C5—C6—C1	−0.66 (18)	C14—C15—C16—C11	0.2 (2)
C4—C5—C6—C7	179.07 (13)	C12—C11—C16—C15	0.75 (19)
N1—C1—C6—C5	179.96 (11)	C9—C11—C16—C15	−177.17 (12)
C2—C1—C6—C5	0.96 (19)	C7—C8—C17—C18	127.05 (15)
N1—C1—C6—C7	0.16 (14)	N1—C8—C17—C18	−54.28 (17)
C2—C1—C6—C7	−178.84 (12)	C7—C8—C17—C22	−54.79 (19)
C5—C6—C7—C8	−179.77 (14)	N1—C8—C17—C22	123.88 (14)
C1—C6—C7—C8	−0.02 (14)	C22—C17—C18—C19	−1.4 (2)
C5—C6—C7—C9	5.8 (2)	C8—C17—C18—C19	176.80 (12)
C1—C6—C7—C9	−174.44 (12)	C17—C18—C19—C20	0.7 (2)
C6—C7—C8—N1	−0.13 (14)	C18—C19—C20—C21	0.2 (2)
C9—C7—C8—N1	174.87 (11)	C19—C20—C21—C22	−0.3 (2)
C6—C7—C8—C17	178.65 (13)	C20—C21—C22—C17	−0.4 (2)
C9—C7—C8—C17	−6.4 (2)	C18—C17—C22—C21	1.24 (19)
C8—C7—C9—C11	102.42 (14)	C8—C17—C22—C21	−176.96 (12)
C6—C7—C9—C11	−83.90 (16)	C2—C1—N1—C8	178.66 (14)
C8—C7—C9—C10	−133.94 (12)	C6—C1—N1—C8	−0.25 (14)
C6—C7—C9—C10	39.74 (17)	C7—C8—N1—C1	0.24 (15)
C7—C9—C10—N2	51.15 (13)	C17—C8—N1—C1	−178.67 (11)
C11—C9—C10—N2	179.61 (9)	C9—C10—N2—O2	53.52 (14)
C7—C9—C11—C12	36.52 (16)	C9—C10—N2—O1	−126.47 (11)
C10—C9—C11—C12	−90.17 (13)		

### (IV) 5-Chloro-3-(2-nitro-1-phenylethyl)-2-phenyl-1H-indole Hydrogen-bond geometry (Å, º)

**Table:**

*D*—H···*A*	*D*—H	H···*A*	*D*···*A*	*D*—H···*A*
N1—H1···O2^i^	0.814 (16)	2.517 (16)	3.0806 (15)	127.4 (14)
C14—H14···O1^ii^	0.95	2.60	3.1827 (17)	120

Symmetry codes: (i) −*x*+1, −*y*+1, −*z*+1; (ii) *x*, *y*−1, *z*.
